# Indoleamine 2, 3-Dioxygenase 1 and CD8 Expression Profiling Revealed an Immunological Subtype of Colon Cancer With a Poor Prognosis

**DOI:** 10.3389/fonc.2020.594098

**Published:** 2020-12-07

**Authors:** Rixin Zhang, Tiegang Li, Weiqi Wang, Wenqiang Gan, Silin Lv, Zifan Zeng, Yufang Hou, Zheng Yan, Min Yang

**Affiliations:** State Key Laboratory of Bioactive Substances and Function of Natural Medicine, Institute of Materia Medica, Chinese Academy of Medical Sciences and Peking Union Medical College, Beijing, China

**Keywords:** colon cancer, tumor microenvironment, immune subtype, immune checkpoint, prognosis

## Abstract

**Background:**

The Immunoscore method, based on the distribution of the quantification of cytotoxic and memory T cells, provides an indicator of tumor recurrence for colon cancer. However, recent evidence has suggested that immune checkpoint expression represents a surrogate measure of tumor-infiltrating T cell exhaustion, and therefore may serve as a more accurate prognostic biomarker for colon cancer. Indoleamine 2, 3-dioxygenase 1 (IDO1), a potent immunosuppressive molecule, has been strongly associated with T-cell infiltration, but it lacks universal prognostic significance among all of the cancer subtypes. Our aim was to elucidate the prognostic significance of the combination of IDO1 and CD8A expression in colon cancer.

**Methods:**

Gene expression and clinical survival data were analyzed using The Cancer Genome Atlas (TCGA) data set and validated using NCBI Gene Expression Omnibus (NCBI-GEO) cohort. Hierarchical clustering, functional enrichment analyses, and immune infiltration analysis were applied to evaluate the distinctive immune statuses in colon cancer risk subgroups stratified by IDO1 and CD8A expression. Moreover, Multivariate Cox regression analysis and Receiver Operating Characteristic (ROC) analyses were conducted to determine the prognostic value of IDO1/CD8A stratification. The IDO1/CD8A classifier may be suitable for use in the prediction of cancer development. It was validated *via* an *in vivo* murine model.

**Results:**

The stratification analysis demonstrated that the colon cancer subtype with the CD8A^high^IDO1^high^* tumor resulted in the worst survival despite high levels of CD8 infiltrates. Its poor prognosis was associated with high levels of immune response, checkpoint genes, and Th1/IFN-γ gene signatures, regardless of CMS classification. Moreover, the IDO1/CD8A stratification was identified as an independent prognostic factor of overall survival (OS) and a useful predictive biomarker in colon cancer. *In vivo* data revealed the CD8A^high^IDO1^high^ group showed strong correlations with late-stage metastasis of colon carcinoma cells and upregulation of immune checkpoints.

**Conclusions:**

The findings indicate that the proposed IDO1/CD8A stratification has exact and independent prognostic implications beyond CD8 T cell alone and CMS classification. As a result, it may represent a promising tool for risk stratification in colon cancer and improve the development of immunotherapies for patients with colon cancer in the future.

## Introduction

In colon cancer, the immune microenvironment is a strong determinant of the clinical prognosis of patients, both in the context of natural disease progression and response to immunotherapy. Based on the level of cytotoxic immune cells, including cytotoxic T lymphocytes and natural killer (NK) cells infiltrating the tumor microenvironment, tumors can be classified as either immunologically active “inflamed” tumors or immunologically inactive “non-inflamed” tumors (1, 2). Early findings indicated that inflamed tumors have a better prognosis than non-inflamed tumors in colorectal, lung, and ovarian cancers, among others (3–5). Immunoscore, a scoring system that summarizes the density of CD3+ and CD8+ T cell effectors within the tumor and its invasive margin, has been suggested to be a better determinant of tumor prognosis (6–8). Likewise, assessment of the immune status *via* the Immunoscore has indicated that is of superior prognostic value compared to the microsatellite-instability (MSI) status and could help guide immunotherapy strategies (9). Recently, an international multicenter study showed that the Immunoscore provided a reliable estimate of the risk of recurrence in patients with colon cancer, which supported establishing a TNM-immune classification as the new prognostic marker (10).

Nevertheless, the evaluation of immune infiltrates is even more complex, not only because of the numerous cell types that can be found in tumors, but also the possibility that immune cells can vary their phenotypes and functions *via* other immune cells or immunosuppressive molecules. Traditionally, the cluster of CD8 T cells is considered to be the key component of effective antitumor immunity (11), as tumors with higher levels of infiltrating CD8 T cells have been associated with improved patient survival (12). However, CD8+ cytotoxic T lymphocytes (CTLs) encounter dysfunction and exhaustion during cancer progression because cancer-associated fibroblasts (CAFs), macrophage type 2 (M2) cells, and regulatory T cells (Tregs) can act as immunological barriers against CD8 T cell-mediated antitumor immune responses (13). Furthermore, the co-expression of programmed death 1 (PD-1) and T-cell immunoglobulin mucin 3 (TIM3) identifies a CD8 T-cell exhaustion phenotype (14). During cancer progression, tumor-infiltrating T cells have been shown to display increased chronic expression of different antagonistic immune checkpoints, causing functional exhaustion and unresponsiveness of T cells (15). The exhausted CD8 T cells failed to proliferate in response to an antigen and lacked critical anticancer effector functions (16–18). Colon cancer occurrence and progression involves multiple aspects of host immune deficiencies. In these events, colorectal cancer (CRC)-associated immune infiltrates can be highly heterogeneous and vary their phenotypes in a spatiotemporal manner (19, 20). Consequently, evaluation of colon cancer patients’ prognosis requires a finer classification of the immunological microenvironment beyond CD8 T cell infiltration.

Indoleamine 2,3-dioxigenase 1 (IDO1) is expressed by various cancer cells and immune cells in the tumor microenvironment, including T cells, dendritic cells (DCs), tumor-associated macrophages, mesenchymal stromal cells (MSCs), and myeloid-derived suppressor cells (MDSCs) (21, 22). As a pivotal contributor to immunosuppression, higher IDO1 expression at the tumor invasion front is involved in CRC progression, and it has been found to correlate with impaired clinical outcomes (23). In addition, T-cell-mediated IDO1 enhancement has been found to correlate with poor glioblastoma prognoses (21). Although functional inactivation of effector CD8 T cells by IDO1 has been established as an important mechanism of immune evasion (24), whether the combination between IDO1 expression and CD8 T cell infiltration could have a beneficial effect on the clinical and prognostic significance for colon cancer cases has not been clarified.

In the present study, we investigated the relationship between IDO1 expression and prognosis of colon cancer in combination with the levels of CD8 T cells using public data sets from The Cancer Genome Atlas (TCGA) and NCBI Gene Expression Omnibus (NCBI-GEO). Moreover, we identified the immune characteristics in colon cancer risk subgroups stratified by IDO1 and CD8A expression and the association of the above risk subgroups and consensus molecular subtypes (CMS). We further validated the correlation of IDO1/CD8A stratification and cancer progression, and investigated tumor immune status through an *in vivo* model. Our study can serve as a theoretical basis for the combined evaluation of IDO1 and CD8A expression, and as a promising prognostic and predictive biomarker for colon cancer.

## Materials and Methods

### Data Resources

RNA-seq data for the TCGA-colon adenocarcinoma (TCGA-COAD) cohort were downloaded using the UCSC Xena browser (http://xena.ucsc.edu/). RNA-Seq by Expectation-Maximization (RSEM) expression values (level 3 RNA-Seq data using IlluminaHi-seq and IlluminaGA platform) were used in this study. The ComBat method was used to normalize the expression values from different batches or platforms in R language with *sva* package (25). Principal component analysis (PCA) was used to visualize the effect of removing inter-batch difference. Clinical data were also obtained using the UCSC Xena browser. We excluded patients without OS information or follow-up time less than 20 days. Data on a total of 438 patients with colon cancer with fully clinicopathological parameters were obtained from the TCGA database. In order to further verify the results, the NCBI-GEO, GSE17538 data set was recruited, and data on a total of 232 patients with colon cancer with complete survival information were included for the following study (https://www.ncbi.nlm.nih.gov/geo/). Information on CMS subtyping calls and sample annotations was downloaded from the Colorectal Cancer Subtyping Consortium Synapse (26).

### Analysis of Immune Infiltration and Immune-Related Genes Expression

The TISIDB database (http://cis.hku.hk/TISIDB) is an integrated repository web portal for the analysis of interactions between tumors and the immune system, and includes literature mining results from the PubMed database and TCGA (27). In this study, we employed the TISIDB database to analyze the relationship between the expression levels of IDO1 and lymphocytes. The correlation between IDO1 and CD274 was analyzed using Gene Expression Profiling Interactive Analysis (GEPIA, http://gepia.cancer-pku.cn/), a web server for data mining based on TCGA and GTEx data provided through differential expression analysis, correlation analysis, survival analysis, similar gene detection, and dimensionality reduction analysis (28). Moreover, the level of immune infiltration across different risk groups for colon cancer was analyzed by Tumor Immune Estimation Resource (TIMER) in the “Estimation” module. TIMER is a web server for analyzing tumor-infiltrating immune cells. Six tumor-infiltrating immune subsets (B cells, CD4 T cells, CD8 T cells, macrophages, neutrophils, and dendritic cells) were evaluated by analyzing 10897 samples of 32 cancer types (29). Here, the abundance of six tumor-infiltrating immune subsets across the risk groups of colon cancer was estimated. Cluster heatmaps of immune checkpoints based on gene expression profiles were generated by the R package ComplexHeatmap (version 2.4.2; https://bioconductor.org/packages/ComplexHeatmap/). The expression pattern of immune-related genes in terms of the expression of immunosuppressive gene sets, cytolytic activity gene sets, and type-1 T helper cells (Th1) signaling gene sets in different subgroup samples was visualized by the R program (Version 4.0.0). Specifically, the expression data set matrix first underwent Z-score normalization according to each gene to best highlight the differences in expression. Then, the samples were hierarchically clustered using Euclidean distance. The correlation plot was generated to show the relationships among immune checkpoints by R package *corrplot* (version 0.84; https://github.com/taiyun/corrplot).

### Functional Enrichment Analysis

To elucidate the potential mechanisms underlying the poor prognosis in group IV*, an enrichment analysis was performed to discover whether there was a potential immune-related regulation pathway that played a role in the pathogenesis and prognosis of patients in this specific group using the gene set enrichment analysis (GSEA) tool (http://www.broadinstitute.org/gsea) (30). Hallmark gene sets and Canonical Pathways gene sets derived from the KEGG pathway database in MSigDB v7.1 were selected for enrichment analysis, and permutations were performed 1,000 times for each analysis. A normalized enrichment score (NES) was calculated for each gene set based on the size of the set. The nominal *P*-value (NOM *P*) of < 0.05 was considered to be significant, and the results obtained by GSEA were plotted on a bubble plot (R package *ggplot*) and volcano plot.

### Animals

Female wild-type C57BL/6J mice at 8 weeks of age were obtained from Beijing Vital River Laboratory Animal Technology Company (Beijing, China) and maintained under specific pathogen-free conditions at the Institute of Materia Medica, Chinese Academy of Medical Sciences, and Peking Union Medical College, China. All of the animal care and experimental protocols complied with the Animal Management Rule of the Ministry of Health, People’s Republic of China (documentation no. 55, 2001) and the Guide for the Care and Use of Laboratory Animals published by the US National Institutes of Health (NIH publication no. 85–23, revised 1996). They were approved by the Institutional Animal Care and Use and Committee of Institute of Materia Medica, Chinese Academy of Medical Sciences, and Peking Union Medical College.

### Mouse Model of Colon Cancer Liver Metastasis

*In vivo* liver metastasis model of colon cancer was performed as described in our previous studies (31–33). Murine colon cancer cell line SL4 cells were maintained in a DMEM/F12 culture medium and supplemented with 10% FBS in a humidified 37°C incubator under 5% CO_2_. Briefly, after a transverse incision in the left flank of an anesthetized mouse was made to expose the spleen, 0.1 ml of a viable cell suspension containing 3 × 10^6^ SL4 cells/mouse was injected into the spleen with use of a 26-G needle. Mice were sacrificed on day 4 and day 12 after cell inoculation. The livers of the animals were immediately removed, washed in ice-cold PBS, and weighed, after which a part of the metastatic focus in the mouse liver was frozen in liquid nitrogen. For experimental metastasis assays, SL4 cells were labeled with firefly luciferase as described previously (32). Bioluminescence from the luciferase expressing SL4 cells was determined at day 4 and 12 post-injection, using the IVIS Spectrum CT System (Xenogen Corp., Alameda, CA, US). Mice were anesthetized by isoflurane (2% vaporized in O_2)_ and imaged 10 min after intraperitoneal injection of D-luciferin solution (150 mg/kg, Caliper Life Sciences, Alameda, CA, US). Bioluminescence intensity plots were quantified as photon flux (p/s/cm2/sr) using LivingImage software (Xenogen Corp.).

### Real-Time PCR

Total-RNA was extracted using the RNeasy kit (Beyotime, Shanghai, China, R0027) according to the manufacturer’s directions. First-strand cDNA was synthesized from an RNA template (1 μg) using SuperScript II reverse transcriptase (TaKaRa, Japan, RR047). Real-time PCR was then performed using the SYBR Green Mix (TaKaRa, Japan, RR820) on an ABI 7900 HT Real-Time PCR system in duplicate. The primer sequences were as follows: IDO1, 5′-GAAAGCTCTTCTGAGTTGGCCT-3′ (forward) and 5′-GATGAAGGTGTTTTCTGTGCCC-3′ (reverse); CD8A, 5′-AGGCCCTCTCCCATGTCTAA-3′ (forward) and 5′- CGGGGGTGCTAAGGAATGTT-3′ (reverse); PD-1, 5′- ACTGCTACTGAAGGCGACAC-3′ (forward) and 5′- AGCCCAAGTGAATGACCAGG-3′ (reverse); programmed death ligand 2 (PD-L2), 5′-ATTGCATGGGCTTTGTGCTC-3′ (forward) and 5′-ACCACGGGCAAGCTTTTATTC-3′ (reverse); TIM3, 5′-TGTGCTCAAGGGGAACTGAC-3′ (forward) and 5′-ACTCTGCCTTCGTATGTCC-3′ (reverse); CD276, 5′-ACCTTGCTTCCGACTTACCC-3′ (forward) and 5′-GGGCCATGCTTTCTCCATGT-3′ (reverse); CD200, 5′-ACCCCAGCTTCTTTTTCTGTGA-3′ (forward) and 5′-TGTCTTCAGAACAAAGCGATTGTA-3′ (reverse); CD160, 5′-AGCCCGTGAACTTTCGTGTA-3′ (forward) and 5′-AGCTCAGTGGCTTCACAAAT-3′ (reverse); GADPH, 5′-TGGAGAAACCTGCCAAGTATGA-3′ (forward) and 5′-GGTCCTCAGTGTAGCCCAAG-3′ (reverse). All of the data were normalized to GADPH mRNA.

### Ethical Statement

The study was approved by the Ethics Committee of Institute of Materia Medica, Peking Union Medical College, and Chinese Academy of Medical Sciences, and abided with the principles of the Declaration of Helsinki. The requirement for informed consent was waived owing to the nature of the retrospective study and the use of publicly available data.

### Statistical Analysis

The optimal cutoff value was determined using the *cutp* function of the R package *survMisc* (version 0.5.5; https://CRAN.R-project.org/package=survMisc), and the expression value corresponding to the highest log-rank test score was utilized to separate patients into high- and low-expression groups with different risks. Upon observing a bimodal distribution of log-rank test score for colon cancer, the IDO1 expression value at a secondary peak was used to identify a second set of high-risk subjects. Kaplan-Meier curves were plotted to compare overall survival (OS), disease-specific survival (DSS), or disease-free survival (DFS), and a log-rank test was performed to estimate the difference between survival statuses. Univariate and multivariate analyses of the Cox proportional hazards regression models were conducted to estimate the hazard ratio (HR) and confidence intervals (CI), and the results were visualized using Prism (version 8.0) as a forest map. Both clinical variables considered as known prognostic factors and significant parameters identified by univariate analysis were included in multivariate analysis, while IDO1 expression was not entered into the multivariate model due to collinearity with the risk groups. Receiver operating characteristic (ROC) curve analysis was performed to evaluate the efficacy of IDO1 combined with other variables to distinguish survival status, the binary logistic regression was utilized to combine multivariates into one index and the area-under-the-curve (AUC) value was calculated and used to designate the ROC effect, where AUC > 0.7 (which indicates higher sensitivity and specificity of diagnosis) and *P* < 0.05 were considered to have certain diagnostic ability. The correlation between the two genes was examined by Spearman’s correlation analysis, the comparison between different risk groups were analyzed by unpaired t-test or Welch’s t-test according to the homogeneity of variance, and Wilcoxon rank sum test was used when the distribution did not meet the normal distribution. A one-way ANOVA was used when more than two risk groups were involved in the test. The Fisher exact test was applied to determine the discrepancy between two groups in the four-fold table due to the total sample size was lower than 40. Statistical analysis was performed using SPSS 25.0 (SPSS Inc., Chicago, IL, US) or Prism (version 8.0). All of the CIs were stated at the 95% confidence level. A *P*-value of < 0.05 was considered to be statistically significant.

## Results

### Association Between IDO1 Expression and Survival in Colon Cancer

**Figure 1:**
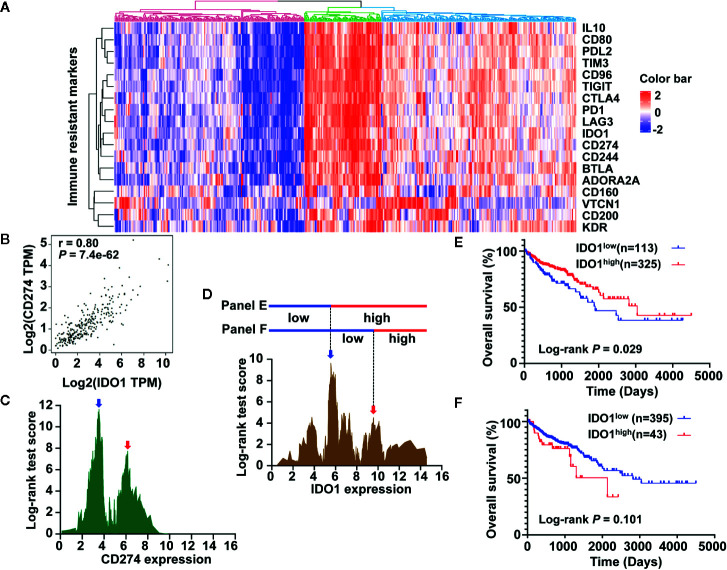
Development of immune prognostic classifier stratified by IDO1 expression in colon cancer patients from the The Cancer Genome Atlas (TCGA) data set. **(A)** The heat map showing gene expression of adaptive immune-resistance markers from the TCGA colon adenocarcinoma (COAD) cohort. **(B)** Correlation between IDO1 and CD274 mRNA expression levels in TCGA COAD samples; r represents the correlation coefficient of Spearman’s analysis. **(C)** The bimodal distribution based on log-rank test across CD274 expression. **(D)** A similar distribution was observed for IDO1, and the expression value of two peaks (shown by the blue arrow and red arrow, approximately 5.561 and 9.576, respectively) was applied to dichotomize patients into high or low expression groups, as shown in **(E, F)**. **(E)** Kaplan–Meier survival curves of OS for low and high IDO1 expression groups dichotomized by the cut-point indicated **(E)**. **(F)** Survival curves of OS based on the distinct cut-off indicated **(F)**. The log-rank test *P* values are shown for each plot.

To obtain a robust transcriptome classification, a total of 438 COAD samples from two independent platforms in TCGA data sets were analyzed. First, the scatter plot based on a PCA of normalized expression revealed that the batch effect produced by different platforms was clearly removed using the ComBat method (**Supplementary Figure S1**). To investigate the underlying immune pattern of colon cancer, the expression of adaptive immune-resistance markers was evaluated using RNA expression data from the TCGA COAD cohort. The heatmap of clustering analysis revealed that the expression of immune-resistance markers presented a large degree of heterogeneity with different levels in colon cancer. We also noticed that the upregulation of IDO1 frequently co-occurred with the upregulation of PD-1, CD274 (also known as programmed death ligand 1, PD-L1), and cytotoxic T-lymphocyte-associated protein 4 (CTLA4) in colon cancer. Notably, the expression of IDO1 in colon cancer showed marked clustering with CD274 (**Figure 1A**). Emerging evidence suggests that the stratification of colorectal cancer microenvironment based on tumor-infiltrating lymphocytes (TILs) and CD274 expression is a biomarker and strong predictor of disease recurrence and survival in patients with CRC (34). As expected, the expression of IDO1 was strongly correlated with CD274 expression in the GEPIA database (**Figure 1B**). A correlation coefficient heatmap showed that IDO1 had a significant positive correlation with immunosuppressive molecules such as CD274 in the TCGA COAD cohort (r = 0.733, *P* < 0.01) (**Supplementary Figure S2**). Specifically, colon cancer presented a different, double-peak pattern based on log-rank tests of CD274 (**Figure 1C**), and the bimodal distribution was also found in IDO1 expression by the TCGA cohort (**Figure 1D**). In the Kaplan-Meier curves, the higher expression of IDO1 indicated improved OS in patients with colon cancer from the TCGA data set (**Figure 1E**) according to the first cut-off value of IDO1 level, which used the highest peak (represented by a blue arrow in **Figure 1D**). Intriguingly, the trend that IDO1 was associated with poor OS in patients with colon cancer was shown (HR = 1.621, 95% CI: 0.801–3.281, *P* = 0.101) (**Figure 1F**) based on the secondary cut-off value of IDO1 level (represented by a red arrow in **Figure 1D**).

### IDO1 Expression Was Correlated With Immune Infiltration Levels, Especially Infiltrating Levels of CD8 T Cells in olon Cancer

**Figure 2:**
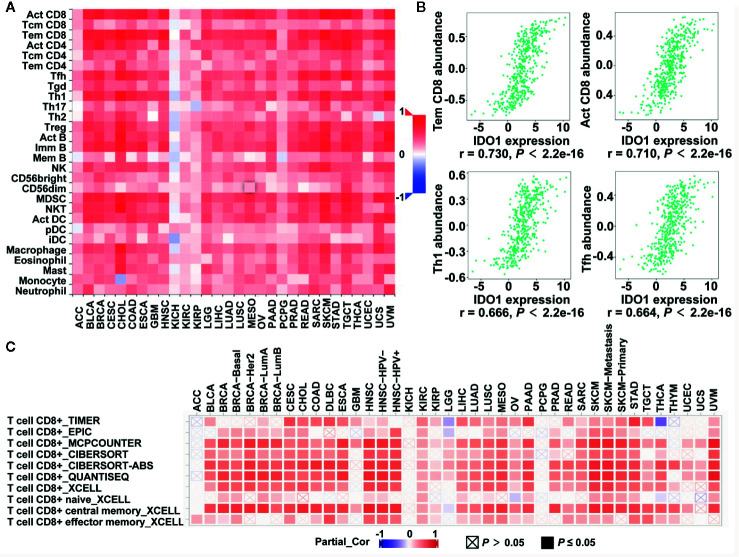
Correlation of IDO1 expression with immune infiltrates level in the The Cancer Genome Atlas (TCGA) cohort. **(A)** The Spearman correlation between the expression of IDO1 and the abundance of 28 TILs across pan-cancer by TISIDB. **(B)** The correlation of IDO1 vs. Tem CD8, Act CD8, Th1, and Tfh for colon adenocarcinoma (COAD) patients from the TISIDB database. The Spearman correlation coefficient (r) and corresponding p value are shown at the bottom of each plot. **(C)** The Spearman correlation between the expression of IDO1 and the CD8+ T cells in different databases across pan-cancer by TIMER2.0. TILs, tumor-infiltrating lymphocytes; Tem CD8, effector memory T cells; Act CD8, activated CD8+ T cells; Th1, type-1 T helper cells; Tfh, T follicular helper cells.

TILs are a prognostic indicator for colorectal cancer (35, 36). To clarify the relationship between IDO1 and immune infiltration levels, we assessed the correlations of IDO1 expression with TIL infiltration levels in the TISIDB database. **Figure 2A** illustrates the important signatures of the 28 types of TILs, including CD8 T cells, CD4 T cells, Th1, type-2 T helper cells (Th2), T follicular helper cells (Tfh), type-17 T helper cells (Th17), regulatory T cells (Treg), B cells, macrophages, monocytes, neutrophils, natural killer cells, dendritic cells, and masts across human heterogeneous cancers. The correlation coefficient between IDO1 and TILs, as well as the corresponding *P* value are listed in **Supplementary Table S1**. IDO1 expression was strongly positively correlated with TILs in many types of human cancer, with the exceptions of several types of kidney cancer, pheochromocytoma and paraganglioma (PCPG), and adrenocortical carcinoma (ACC) (**Figure 2A**). The Spearman’s correlation showed that IDO1 was strongly related to immune infiltration in COAD, especially the four most significant infiltrators of immune cells: effector memory T cells (Tem CD8, r = 0.730), activated CD8+ T cells (Act CD8, r = 0.710), Th1 (r = 0.666), and Tfh (r = 0.664) (**Figure 2B**). Additionally, the TIMER2.0 online databases were employed to further analyze the relationship between IDO1 and the marker genes of CD8 T cells in the pan-cancer profiling (**Figure 2C** and **Supplementary Table S2**). In line with TISIDB, IDO1 was remarkably correlated with the abundance of CD8 T cells among human malignancies such as cervical squamous cell carcinoma (CESC), head and neck squamous cell carcinoma (HNSC), skin cutaneous melanoma, and COAD (**Figure 2C**). The comprehensive and detailed analysis of the relationship between IDO1 and immune cells in various databases among colon cancer and different tumors may indicate that IDO1 is strongly linked to immunological properties in the tumor microenvironment, especially the levels of CD8 T cells.

### Identification and Validation of Colon Cancer Risk Subgroups Defined by IDO1 and CD8A Expression

**Figure 3:**
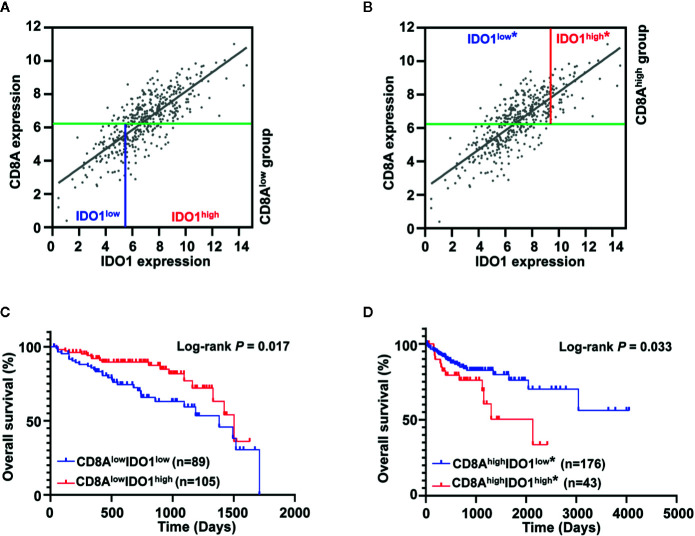
Prognostic significance of IDO1 was dependent on CD8A gene expression levels in the The Cancer Genome Atlas (TCGA) colon adenocarcinoma (COAD) cohort. **(A, B)** Scatter plots for the expression between IDO1 and CD8A were shown, and the median expression value of CD8A (indicated with green solid line) was applied to divide patients into CD8A^low^ group and CD8A^high^ group. The highest log-rank test score of IDO1 expression in each group was applied to further dichotomize the patients (CD8A^low^ group in a solid blue line; CD8A^high^ group in a solid red line). **(C, D)** Kaplan–Meier survival curves for the risk groups stratified by optimal IDO1 cut-offs and median expression value of CD8A shown **(A, B)**, 5-year survival curves are shown for the CD8A^low^ group and survival curves across overall time are shown for the CD8A^high^ group. The log-rank test *P* values are shown for each plot. "*" in the figures indicate that this stratification is according to the secondary IDO1 peak.

Previous studies showed that the bimodal distribution of CD274 expression led to the opposite survival pattern in CRC, stratified by a different cut-off value of CD274 expression (34). Although IDO1 expression was associated with the gene signature of CD8 T cells in colon cancer, the different expression patterns of IDO1 and CD8A imply biologically and clinically different behavior. Therefore, we examined whether prognostic significance of IDO1 could be dependent on CD8A gene expression levels in colon cancer. **Figure 1D** presents the bimodal distribution of log-rank test scores for IDO1 expression. For further analysis, colon cancer patients were divided into two groups, with low expression of CD8A and high expression of CD8A, according to the median value of CD8A (**Figures 3A, B**). This was followed by OS analysis based on the optimal cut-off value of IDO1 expression in each subgroup. Stratification analysis demonstrated that high IDO1 expression was associated with improved OS in patients with colon cancer in the absence of CD8A expression (**Figure 3C**), whereas increased IDO1 levels in colon cancer correlated with poor prognosis with high CD8A expression subgroup (HR = 2.003, 95% CI: 0.916–4.380, *P* = 0.033) (**Figure 3D**). These findings suggest that the opposite IDO1 prognostic outcomes are dependent on CD8A gene expression levels.

**Figure 4:**
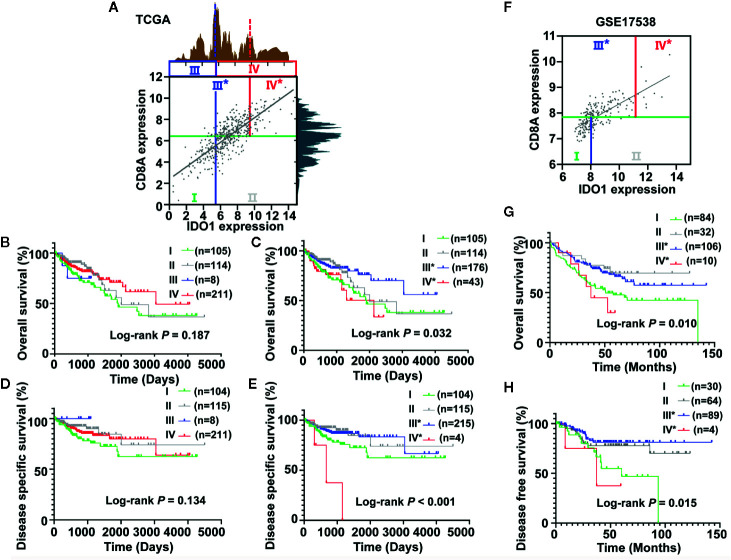
Identification and validation of the colon cancer risk subgroups based on the combined analysis of IDO1 and CD8A expression. **(A)** Scatter plot showing the expression between IDO1 and CD8A in colon cancer from The Cancer Genome Atlas (TCGA). Colon cancer patients were stratified into four groups based on bimodal value of IDO1 and the median expression value of CD8A. The groups divided by the first IDO1 peak (using solid blue lines) were named Group I, II, III, and IV. The groups were named Group I, II, III*, IV* when separated according to the secondary IDO1 peak (the higher cut-off for IDO1 expression, using solid red lines). Survival curves of OS **(B, C)** and DSS **(D, E)** across the colon cancer risk groups stratified by different IDO1 peaks were compared by Kaplan–Meier analysis for the TCGA cohort. **(F)** Scatter plot showing the expression between IDO1 and CD8A in colon cancer from the GSE17538 data set. The validation cohort (GSE17538) was conducted using the same approach to identify group III* and group IV* using the secondary IDO1 peak (the higher cut-off for IDO1expression). Kaplan–Meier survival plots for OS **(G)** and DFS **(H)** stratified according to the combined the secondary IDO1 peak and the median expression value of CD8A in the GSE17538 data set. The log-rank test *P* values are shown for each plot. OS, overall survival; DSS, disease specific survival; DFS, disease free survival.

To demonstrate the existence of the colon cancer risk subtypes, we applied the highest IDO1 peak (shown as a dotted blue line in **Figure 4A**) or the secondary IDO1 peak (the higher cut-off for IDO1expression, shown as a dotted red line in **Figure 4A**) to define the IDO1^high^ and IDO1^low^ populations, respectively, and then carried out stratification based on the median value of CD8A expression. According to the expression groups of IDO1 combined with CD8A, we divided the TCGA COAD patients into four combinations in terms of the highest IDO1 peak and the median value of CD8A, denoted as group I (CD8A^low^IDO1^low^), group II (CD8A^low^IDO1^high^), group III (CD8A^high^IDO1^low^), and group IV (CD8A^high^IDO1^high^). Then, we applied this secondary cut-off (the higher cut-off for IDO1expression, shown as a dotted red line in **Figure 4A**) for stratifying the new group III* (CD8A^high^IDO1^low^*) and the new group IV* (CD8A^high^IDO1^high^*). The survival analysis of these four groups showed that no significant association between the risk groups stratified by the highest IDO1 peak and the median value of CD8A and clinic outcome for colon cancer was seen (**Figure 4B**). Interestingly, we observed the lowest OS in group IV*, even though it had high levels of CD8A, whereas Group III* had the most favorable outcomes for colon cancer with higher CD8A expression in the absence of IDO1 expression (*P* = 0.032) (**Figure 4C**). Similar trends toward DSS were also observed when the TCGA COAD cohort was stratified in line with the same method (**Figures 4D, E**). To further explore the prognostic value of the subgroup analyses according to IDO1 and CD8A expressions, Kaplan-Meier survival analysis from the validation cohort (GSE17538) was conducted. We used the same approach to identify group III* and group IV* using the secondary IDO1 peak (the higher cut-off for IDO1expression) (**Figure 4F**). Consistent with the TCGA COAD cohort, group IV* had significantly worse OS (*P* = 0.010) (**Figure 4G**) and DFS (*P* = 0.015) (**Figure 4H**) than group III* did from GSE17538.

**Figure 5:**
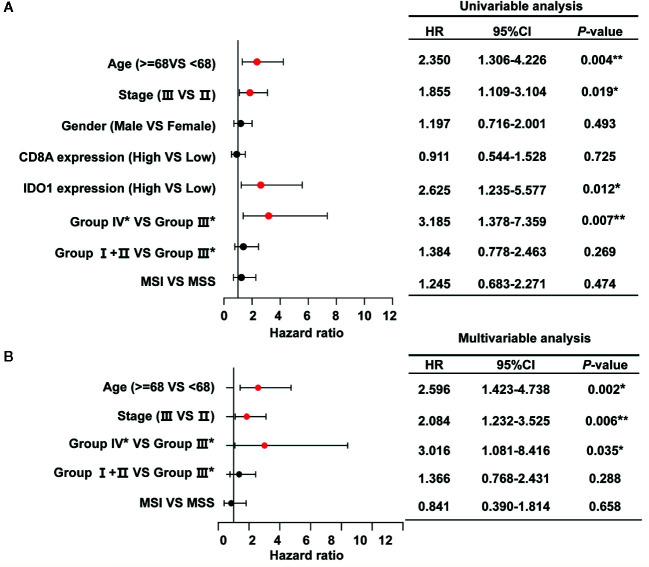
Univariate **(A)** and multivariate **(B)** analysis for OS calculated by Cox proportional hazard analysis based on the stage II and stage III patients (n=295) in the The Cancer Genome Atlas (TCGA) colon adenocarcinoma (COAD) cohort. The solid red dots in the forest map represent the clinicopathologic variable that was considered to be significant (*P* < 0.05). **P < 0.01; *P < 0.05. HR, hazard ratio; 95% CI, 95% confidence interval.

Furthermore, we performed univariate and multivariate Cox regression analyses based on stage II and stage III patients, accounting for the majority of the total to identify risk factors correlated with the prognosis of colon cancer. The forest plots showed the significant association of group IV* (CD8A^high^IDO1^high^*) with OS in colon cancer patients from the TCGA data set (**Figure 5A**), and further indicated that group IV* (CD8A^high^IDO1^high^*) serve as an independent predictor of patients’ survival outcome in the TCGA COAD cohort (HR = 3.016, 95% CI: 1.081-8.416, *P =* 0.035) (**Figure 5B**).

### Immune Signatures of the Colon Cancer Risk Subgroups Stratified by IDO1 and CD8A Expression

**Figure 6:**
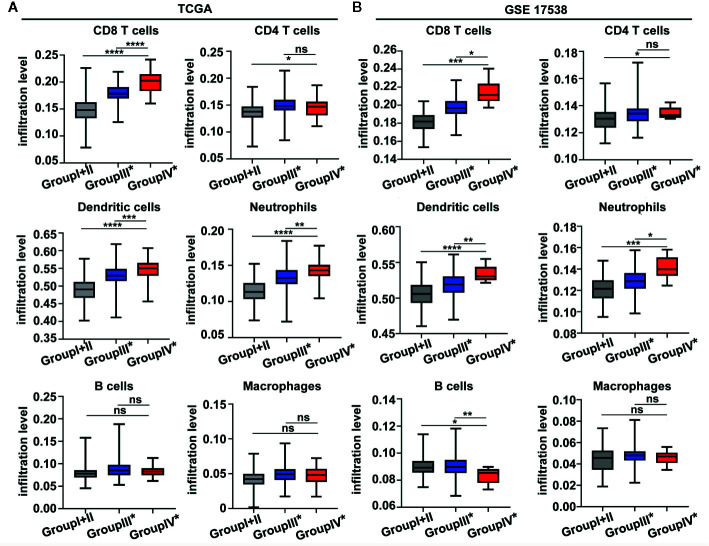
The boxplot shows the immune infiltration level across the colon cancer risk groups by TIMER from the The Cancer Genome Atlas (TCGA) **(A)** and GEO GSE 17538 **(B)** data sets. ****P < 0.0001; ***P < 0.001; **P < 0.01; *P < 0.05; ns, not significant.

**Figure 7:**
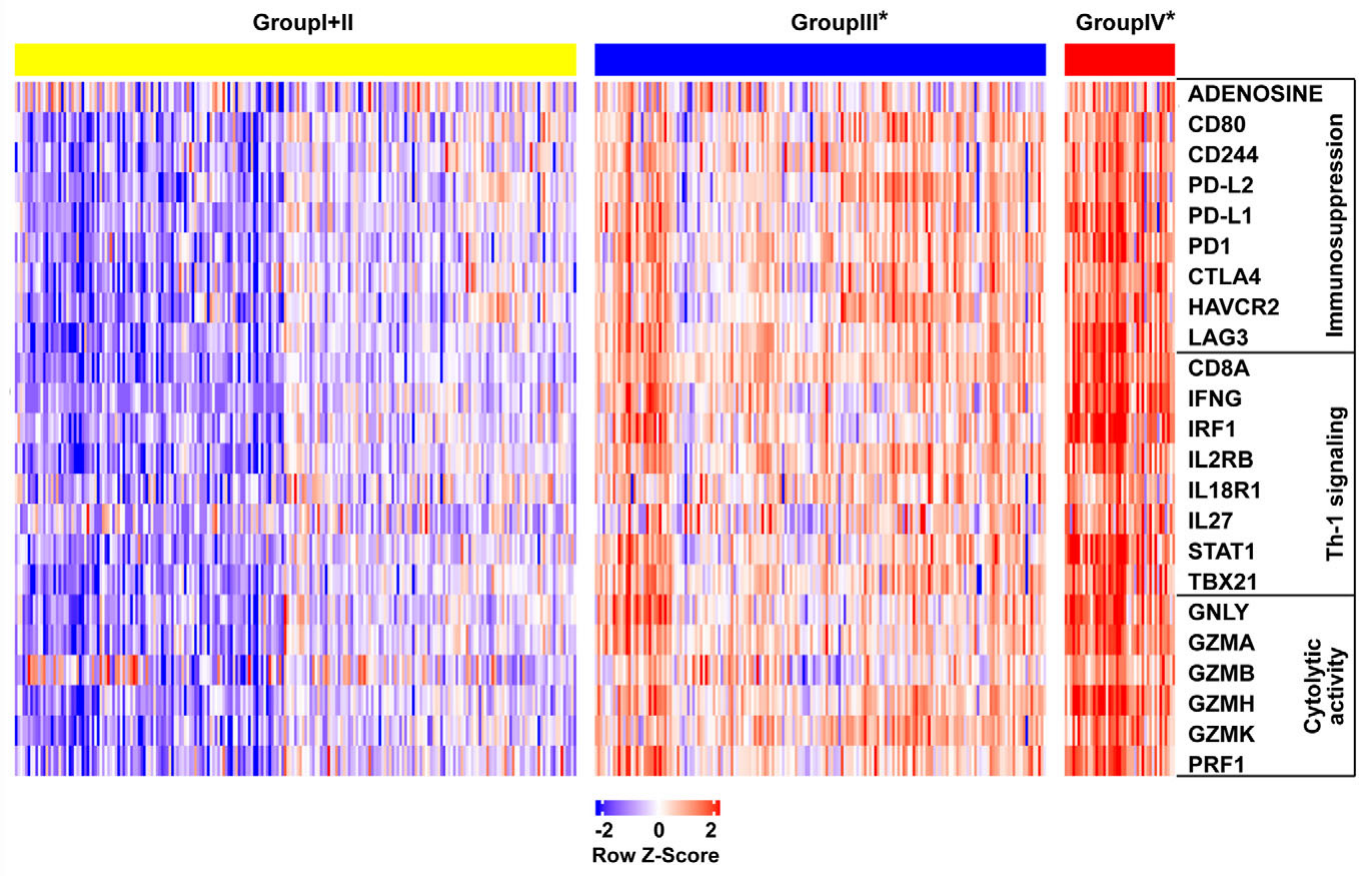
Heat map demonstrating the gene expression profile across the colon cancer risk groups in the The Cancer Genome Atlas (TCGA) cohort. The genes are grouped by their function as immunosuppression, Th-1 signaling, and cytolytic activity. Each column is a sample, each row is a gene.

To investigate the relative proportions of immune infiltrates in the colon cancer risk subgroups, we compared the abundance of immune cell subpopulations including CD8+ T cells, CD4+ T cells, dendritic cells, neutrophils, B cells, and macrophages in the TIMER database. As illustrated in **Figure 6A**, the levels of infiltrating CD8 T cells, dendritic cells, and neutrophils were significantly higher in group IV* (CD8A^high^IDO1^high^*) than in group III* (CD8A^high^IDO1^low^*) or group I+II (CD8A^low^). Consistent with the TCGA COAD cohort, the GSE17538 data set showed that the same three major cell-type infiltrates were highly enriched in group IV* (**Figure 6B**). Specifically, immune checkpoint genes, including CD274, lymphocyte-activation gene 3 (LAG3), TIM3, T cell Ig, and ITIM domain (TIGIT), were all markedly upregulated in the group IV* samples in both the TCGA and GSE17538 data sets (**Supplementary Figure S3A, B**). In contrast, group IV* had relatively lower levels of KRT20 expression (the cancer cell marker) than group III* or group I+II (**Supplementary Figure S3C**). We then performed a clustering analysis of immune-related genes for the colon cancer risk subgroups stratified by CD8A and IDO1 using the TCGA data. As shown in the heatmap (**Figure 7**), the multigene signatures of the immune checkpoints exhibited the highest expression in the group IV* samples. Likewise, the expression levels of Th-1 signature genes were the most highly increased in group IV* compared with the other subgroups, and a similar co-expression pattern of cytotoxic molecules was also observed across the colon cancer risk subgroups (**Figure 7**). To gain further insight into the biological pathways involved in the colon cancer risk subgroups, we performed a gene enrichment analysis between group IV* and group III* samples. The bubble plot and volcano plot showed the enriched biological pathways based on the NES values in the group IV* samples (**Supplementary Figure S4A, B**). The gene signatures implied that the most significantly altered pathways, those involving reactive oxygen species, interferon gamma/alpha response, antigen processing and presentation, the toll-like receptor signaling pathway, natural killer cell–mediated cytotoxicity, IL6-JAK-STAT3, and T cell receptor signaling pathway were significantly enriched in group IV* samples with high CD8A and IDO1 expression (**Supplementary Figure S4A, B**). Enrichment plots of GSEA further demonstrated group IV* was highly associated with the signatures of adaptive immune response and T cell-mediated immunity (**Supplementary Figure S4C**).

### Association Between CMS Status and Colon Cancer Risk Subgroups Stratified by IDO1 and CD8A Expression

**Figure 8:**
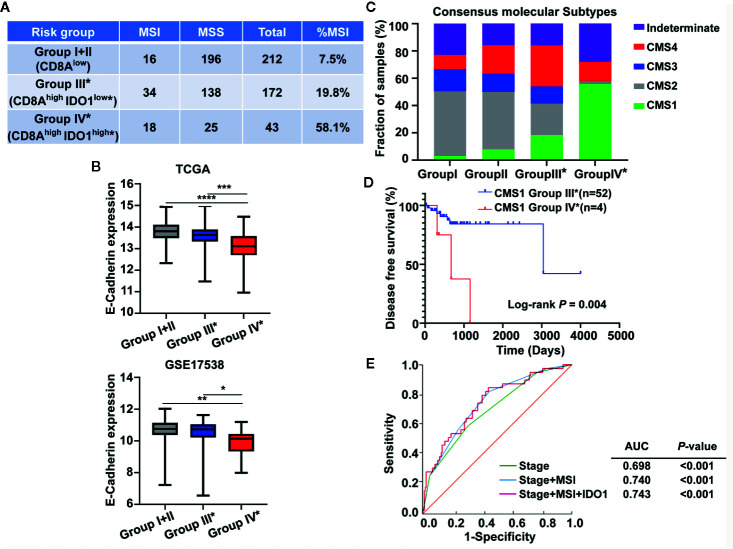
Association between the CMS status and the IDO1/CD8A-stratified colon cancer risk groups in the The Cancer Genome Atlas (TCGA) cohort. **(A)** The microsatellite instability status across the colon cancer risk groups. **(B)** Comparison of E-cadherin expression levels across the colon cancer risk groups from the TCGA and GEO GSE 17538 data sets. ***P < 0.001; **P < 0.01; *P < 0.05. **(C)** The proportion of CMS subtype (CMS1, MSI immune; CMS2, canonical; CMS3, metabolic; CMS4, mesenchymal) in each of the stratified risk groups. **(D)** Kaplan–Meier survival curves comparing the CD8A^high^ risk groups III* and IV* in CMS1 patients for DSS. The log-rank test *P* value is shown for the plot. **(E)** ROC curves illustrate the sensitivity and specificity for different variables: stage alone, stage combined with MSI status and plus the expression of IDO1 in predicting overall survival of the CD8A^high^ risk groups. MSI, microsatellite-instability; DSS, disease specific survival; ROC, receiver operating characteristic; AUC, area under the curve.

Given that CMS1 is heavily enriched for MSI tumors featuring immune infiltration and activation (26, 37, 38), we investigated the MSI status across the colon cancer risk subgroups. As shown in **Figure 8A**, groups IV* and III* with high CD8A expression showed a larger proportion of MSI samples compared with group I+II in the absence of CD8A, and the higher MSI fraction was particularly remarkable in group IV* (CD8A^high^IDO1^high^*). Studies have proposed that CMS4 is a mesenchymal subtype that involves the upregulation of EMT pathways with poor clinical outcomes (26, 37, 38). Interestingly, the expression levels of E-Cadherin (the marker of epithelial cell) were significantly downregulated in group IV* compared with those in other risk groups from the TCGA and GSE17538 data sets (**Figure 8B**). Then, we conducted CMS classification in the context of the colon cancer risk subgroups because the IDO1/CD8A-stratified risk groups were different from the CMS classification. As illustrated in **Figure 8C**, group IV* had a large proportion of CMS1 (MSI immune subtype) and CMS4 (mesenchymal subtype), but was especially overrepresented with CMS1. Moreover, the Kaplan–Meier and log-rank tests revealed that group IV* patients in CMS1 had significantly lower DSS than group III* patients in CMS1 (HR = 5.703, 95% CI: 0.5016–64.83, *P* = 0.004) (**Figure 8D**). To determine the prognostic accuracy of IDO1 in the patients with high CD8A expression, a ROC analysis was conducted, with various combinations of parameters in group III* and group IV* patients. The AUC for overall survival of the prediction model, including the clinical stage, MSI status, and IDO1 expression, significantly improved from 0.698 to 0.743 (**Figure 8E**). The combination index showed an additive predictive value for overall survival compared with the known prognostic factor (**Figure 8E**), hinting at the potential of the prognostic accuracy based on IDO1 for colon cancer patients with CD8A^high^ tumors.

### Correlations of IDO1/CD8A Stratification With Tumor Development and Tumor Microenvironment in a Liver Metastasis Model of Colon Cancer

**Figure 9:**
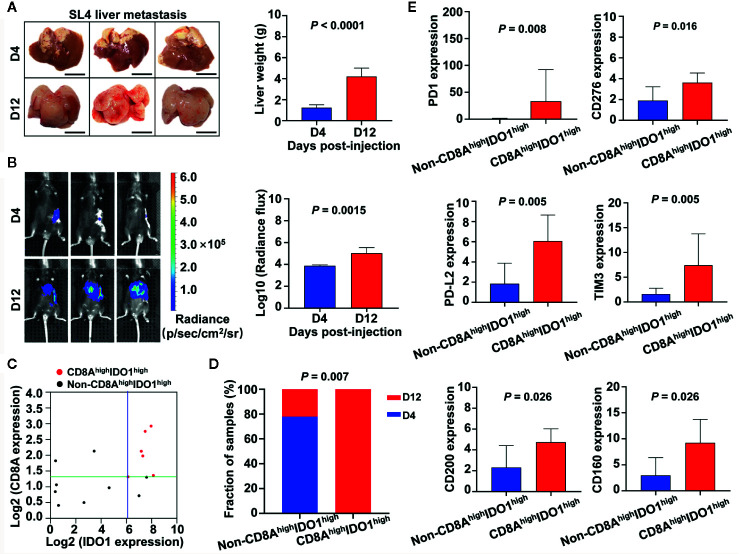
Associations between IDO1/CD8A stratification and cancer progression as well as tumor immune status in liver metastasis model of colon cancer. **(A)** Gross examination of hepatic tumor metastasis of colon cancer after intrasplenic injection of SL4 cells in mice. Mice were sacrificed on day 4 or day 12 after inoculation, and livers were excised, photographed, and weighted. Scale bars=1 mm. Data are mean ± SD for n=6 mice. D indicates day. **(B)** *In vivo* quantification of tumor metastasis by bioluminescence tracking of luciferase expressing SL4 cells at day 4 and 12 post-injection. Histogram showing the bioluminescent signal intensity detected using the IVIS Spectrum CT System. **(C)** Scatter plot showing the expression between IDO1 and CD8A in liver metastatic foci at day 4 and 12 post-injection. All mice were divided into four groups based on the median value in IDO1 and CD8A levels. **(D)** The stacked bars illustrating the proportion of day 4 or day 12 post-injection in CD8A^high^IDO1^high^ group and non-CD8A^high^IDO1^high^ groups, the *P* value of the Fisher exact test is shown in the figure. **(E)** Quantification of immune checkpoint genes mRNA expression levels by real-time PCR. The exact *P* value is shown for each plot.

To further explore associations between IDO1/CD8A stratification and cancer progression or tumor immune status in colon cancer, we utilized a murine model in which mouse colon cancer cells (SL4) were injected into the spleen and developed into liver metastasis. Gross inspection showed multiple hepatic tumor nodules and increased tumor-occupied liver weight in WT mice between day 4 and day 12 after SL4 injection (**Figure 9A**). To quantify metastatic potential *in vivo*, SL4 cells were labeled with firefly luciferase and injected into the pancreases of WT mice. Bioluminescence imaging showed signals from the group at day 12 post-injection were significantly stronger than those from the group at day 4 post-injection (*P* = 0.0015) (**Figure 9B**). Quantitative RT-PCR revealed the mRNA expression of IDO1 and CD8A in liver metastatic foci both at the later point in time (day 12) and the early point in time (day 4) (**Figure 9C**). To assess the potential contribution of high expression of IDO1 with high expression of CD8A in tumor development, all mice given intrasplenic injection of SL4 cells were divided into four groups according to the median value in IDO1 or CD8A levels as follows: CD8A^low^IDO1^low^, CD8A^low^IDO1^high^, CD8A^high^IDO1^low^, and CD8A^high^IDO1^high^ (**Figure 9C**). Given the small number of subgroups, CD8A^high^IDO1^high^ samples were compared to all of the remaining samples in the following analysis. As shown in **Figure 9D**, the proportions of liver metastatic foci at day 12 in the CD8A^high^IDO1^high^ group and the non-CD8A^high^IDO1^high^ group were 100% (6/6) and 22.22% (2/9), respectively, and significantly higher rates of the advanced metastasis were found among the mice in the CD8A^high^IDO1^high^ than in the mice in the non-CD8A^high^IDO1^high^ group (*P* = 0.007). Moreover, other immune checkpoints such as PD-1, PD-L2, TIM3, CD276, CD200, and CD160, were significantly overexpressed in the CD8A^high^IDO1^high^ group compared to the non-CD8A^high^IDO1^high^ group (**Figure 9E**). These results indicate that the upregulation of IDO1 and CD8A expression in the host tumor microenvironment, may contribute to tumor development by association with the increase of immune checkpoints.

## Discussion

Recent evidence has suggested that CD8 T cells with cytotoxic activity play an important role in anti-tumor immunity, and a higher density of CD8+ T cells is associated with a favorable prognosis in a variety of malignant tumors, including CRC (3, 39, 40). Several large CRC studies have indicated that the Immunoscore can lead to a better determination of tumor prognosis (7–10, 41, 42). In particular, the immune contexture is defined as the type, functional orientation, density, and location of adaptive immune cells within distinct tumor regions, which have been shown to have a prognostic value that may be superior to the TNM classification (43). In this regard, several studies have pointed out that the immune checkpoint expression may contribute to immunosuppression and be associated with poor prognosis of CRC despite high CD8 T cell infiltration (34, 44).

Colon cancer is characterized by extreme heterogeneity due to histopathological differences, molecular characteristics, genomic instability, and expression signature of immune genes (45). The recent success of checkpoint inhibition for cancer immunotherapy has generated tremendous enthusiasm for cancer prevention by directly targeting the immune system using immune modulating agents. Whereas blocking antibodies against PD-1, PD-L1, and CTLA-4, known as immune checkpoint inhibitors, have exhibited clinical efficacy in deficient mismatch repair (MMR) or in highly microsatellite instable (MSI-H) colorectal cancer, the vast majority of patients with proficient MMR and with microsatellite stable (MSS) tumors do not benefit from immunotherapy (46–48). Further studies are needed to identify multiple immune biomarkers for a stratification of patients who would most likely benefit from novel immunotherapies. In the present study, unsupervised hierarchical clustering analyses revealed that upregulation of immune-resistance markers, including PD-L1(CD274), was observed only in a subset of colon cancer from TCGA RNA-Seq data. Furthermore, upregulation of CD274 frequently co-occurred with upregulation of other immunosuppressive molecules. The expression of IDO1 in colon cancer in particular showed marked clustering with CD274. Recently, Fakih et al. showed opposite CD274 prognostic behaviors in CRC patients to be dependent on CD8A gene expression levels due to the bimodal distribution of CD274 expression, indicating that the combination of CD8 T cell infiltration with CD274 expression may be a strong predictor of survival in CRC patients (34). Such bimodal distribution was also observed for IDO1 expression among these immune checkpoints. Nonetheless, further evaluation of patients’ prognoses and immunotherapy efficacy beyond the contributions from T cell infiltration, PD-1/PD-L1 and CTLA4 in colon cancer is necessary. Given high inter-patient heterogeneity and high intra-tumor heterogeneity in colon cancer, our study may be useful for stratification of colon cancer and for predicting prognosis and developing novel cancer immunotherapies. In this study, we identified the immunological subtypes of colon cancer based on the expression of IDO1 in combination with CD8A expression, and explored the prognostic values, phenotypes, and functions of distinct subgroups in colon cancer.

IDO1 is recognized as an important mediator of immunosuppression in cancer, and higher IDO1 expression has been found to play important role in immune escape and tumors resistant to immunotherapy (49–51). To evaluate relationships between IDO1 expression and tumor-infiltrating T lymphocytes, Spearman’s correlations between IDO1 expression and lymphocytes were analyzed *via* the TISIDB and TIMER databases. The results showed that IDO1 had the greatest correlation with CD8 T cells. Largely, colon cancer has only a limited response rate to systemic immunotherapies due to its high heterogeneity. Thus, an improved understanding of the colon cancer tumor microenvironment from identifying subsets of patients who may be paired on the basis of immune biomarkers in immunotherapy-responsive state, is needed to improve upon current immunotherapies. Accumulating evidence suggests that the coassessment of immune checkpoint expression and CD8 T cell density may provide a better identification of the immunologic state of solid tumors. In turn, it provides a more accurate prognosis than the Immunoscore approach alone (34, 52, 53). Specifically, our data showed IDO1 expression exhibited a markedly bimodal score distribution in colon cancer. Another important finding was that low and high IDO1 expression groups dichotomized by the distinct cut-points displayed a reverse pattern of survival trends in colon cancer. Therefore, we hypothesized the existence of a novel risk group in colon cancer based on IDO1 expression and lymphocyte infiltration. However, the prognostic value of IDO1 in the tumor microenvironment of colon cancer and underlying immunological mechanisms remains unclear. Given the strong evidential basis for efficacy of IDO1 and CD8+ T cell infiltration in tumor progression and immune escape of colon cancer, we further explored the prognostic relevance of IDO1 expression in combination with CD8A expression. Survival analysis revealed that patients with colon cancer with CD8A^high^IDO1^high^ tumors had worse prognoses than patients with CD8A^high^IDO1^low^ tumors, illustrating that not all patients with high CD8 T cell infiltration have favorable outcomes. Hence, the association of IDO1 expression with CD8 T cell density may be more important than CD8 T cell infiltration alone in predicting survival.

Recently, the analysis of the local CD8 and IDO1 expression profile has been found to serve as a helpful tool in predicting the prognosis of patients with locally advanced rectal cancer following neoadjuvant chemoradiation, with the subgroup of high total IDO1 and CD8 scores having the best prognosis by immunohistochemistry (54). However, the clinical significance of IDO1 expression within primary colon cancer tumors, independent of neoadjuvant radiotherapy and/or chemotherapy, remains incompletely understood. In our data analysis, we found that group IV* (CD8A^high^IDO1^high^*) using the secondary IDO1 peak (the higher cut-off for IDO1 expression) had the worst prognosis among the four subgroups of colon cancer. Consistent with the TCGA findings, the stratified analysis using IDO1 and CD8A expression revealed a similar trend of worse prognosis for group IV* (CD8A^high^IDO1^high^*) using the independent cohort of colon cancer from NCBI-GEO data set. Technical variation and different analysis criteria may potentially result in the opposite prognostic behaviors of IDO1 expression patterns. Therefore, we concluded that any discrepancies between our analysis and these other reports may be predominantly a result of the combinational analysis of IDO1 and CD8A expression and the higher cut-off for IDO1 expression defined in group IV* (CD8A^high^IDO1^high^*) and group III* (CD8A^high^IDO1^low^*).

To further investigate why group IV*(CD8A^high^IDO1^high^*) has poor outcome despite high CD8 T cell infiltration, we analyzed cancer immune phenotypes. Group IV* exhibited “inflamed tumors” with abundant TILs, including CD8 T cells, dendritic cells, neutrophils, and active immune-regulatory pathways. Furthermore, our analysis showed that group IV* tumors had significantly higher levels of immune checkpoints known to inhibit the anti-tumor immune response. Moreover, CD8 T-cells produce interferon (IFN)-γ, which leads to the upregulation of adaptive immune resistance pathway gene expression, including PD-1/PD-L1 and IDO1 (55–57). Our study showed that group IV* tumors had high levels of Th1/IFN-γ gene signatures and cytolytic enzyme expression despite the high expression of multiple immunoinhibitory factors. These results were consistent with those of recent studies, which showed that high levels of Th1 signaling in the TME was associated with poor survival (34, 58). It was tempting to speculate that the tumor-immunosuppressive microenvironment can drive the loss of effector functions on CD8 T cells through their sustained IFN-γ production, which may promote IDO1 expression in CRC, as has been recently reported (59). The process may contribute to diverting CD8 T cell immunity from anti-tumor features to tumor immune escape. This may thereby offer an explanation as to why the group IV* tumors were not associated with favorable prognosis despite their high CD8 T cell infiltration.

CRC has been classified into four CMS subtypes (26), namely, CMS1 (microsatellite instability immune), CMS2 (canonical), CMS3 (metabolic), and CMS4 (mesenchymal). Drawing on the results showing that the higher MSI proportion and E-Cadherin downregulation were particularly enriched in group IV*, we explored whether there was an association between the CMS and colon cancer risk subgroups stratified by IDO1 and CD8A expression using the TCGA data. In the present study, the findings suggest that IDO1/CD8A stratification has additional and independent prognostic implications beyond CMS classification, which may contribute to improved risk-stratification in colon cancer. Using the murine model of colon cancer liver metastasis as shown in our previous studies (31–33), we observed that the advanced metastatic behavior mainly existed in the CD8A^high^IDO1^high^ group with overexpression of some immune checkpoints, but not in the other risk group. Despite the high proportion of CD8 T cells in the tumor microenvironment, tumor cells can still escape from immunology attack due to the immunosuppressive function of IDO1. Furthermore, this effect may be mediated, in-part, through upregulation and enhanced participation of immunosuppressive T-cell impairing ligands, namely, PD-1 and PD-L2 (60). These data led to the discovery of IDO1 as the powerful driver of tumor development *via* establishment of tumor microenvironments of metastasis.

This study had a few limitations. First, only transcriptomic expression of IDO1 and CD8A expression with clinical data was analyzed to predict prognosis in the colon cancer risk subgroups from public databases (TCGA and NCBI-GEO). Our study was the lack of validation with immunohistochemistry assay because we did not have enough Formalin-Fixed and Paraffin-Embedded (FFPE) tissues with intact follow-up data and without treatment to build a large-scale colon cancer cohort. Second, limitations included the retrospective nature of our analyses and selection biases inherent in the cohorts, such as the possible treatment-related effects on the tumor microenvironment. Third, the underlying mechanisms of the distinct prognosis in colon cancer risk subgroups remain unclear, even though several functional annotations and enrichment analysis were conducted. Future research is required to explore the detailed mechanism between the combined IDO1 and CD8A prognostic classifier and tumor development of colon cancer. Despite these limitations, to the best of our knowledge, this study was the first to focus on the IDO1-CD8A gene signature of colon cancer on the basis of population databases for exploration and validation. Moreover, it was closely connected with the prognosis, prediction value, and tumor immune status of patients with colon cancer.

In conclusion, we developed and validated a prognostic molecular classifier based on the evaluation of IDO1 expression and CD8 T cell infiltration. Our study demonstrated that patients with colon cancer with group IV* (CD8A^high^IDO1^high^*) tumors had poor clinical outcomes, even though they had high CD8 T cell infiltration. Thus, the combination of factors could have more promising prognostic and predictive potential of tumor recurrence and OS than CD8+ TIL density alone or CMS classification. Our results highlighted the extent of heterogeneity of colon cancer with respect to immunity, suggesting that the prediction of colon cancer patient outcomes through evaluation of immune components in the tumor microenvironment can likely be improved by integrating immune checkpoint markers, and it may enhance the personalized treatment of colon cancer patients with immune checkpoint inhibitors.

## Data Availability Statement

The original contributions presented in the study are included in the article/**Supplementary Material**, further inquiries can be directed to the corresponding author.

## Ethics Statement

The animal study was reviewed and approved by The Ethics Committee of Institute of Materia Medica, Peking Union Medical College, and Chinese Academy of Medical Sciences. 

## Author Contributions

The study concept and design were done by MY and RZ. Administrative support was provided by MY. Provision of study materials or patients was done by RZ and WW. Collection and assembly of data were done by RZ, WW, and TL. Analysis and interpretation of data were done by RZ, TL, WW, and MY. The mice model was created by WG, SL, and TL. Real-time PCR was done by ZZ and WW. Statistical advice and technical support were provided by YH and ZY. Funding support was provided by MY. All authors contributed to the article and approved the submitted version.

## Funding

This work was supported by the Natural Science Foundation of China (NSFC) Grant (No. 81773750).

## Conflict of Interest

The authors declare that the research was conducted in the absence of any commercial or financial relationships that could be construed as a potential conflict of interest.
