# Supplementary material for: Indoleamine 2, 3-Dioxygenase 1 and CD8 Expression Profiling Revealed an Immunological Subtype of Colon Cancer With a Poor Prognosis
Source: Front Oncol. 2020 Dec 7;10:594098. doi: 10.3389/fonc.2020.594098 (PMC7793995; doi:10.3389/fonc.2020.594098)
Supplement: Supplementary file 1 [file DataSheet_1.pdf]

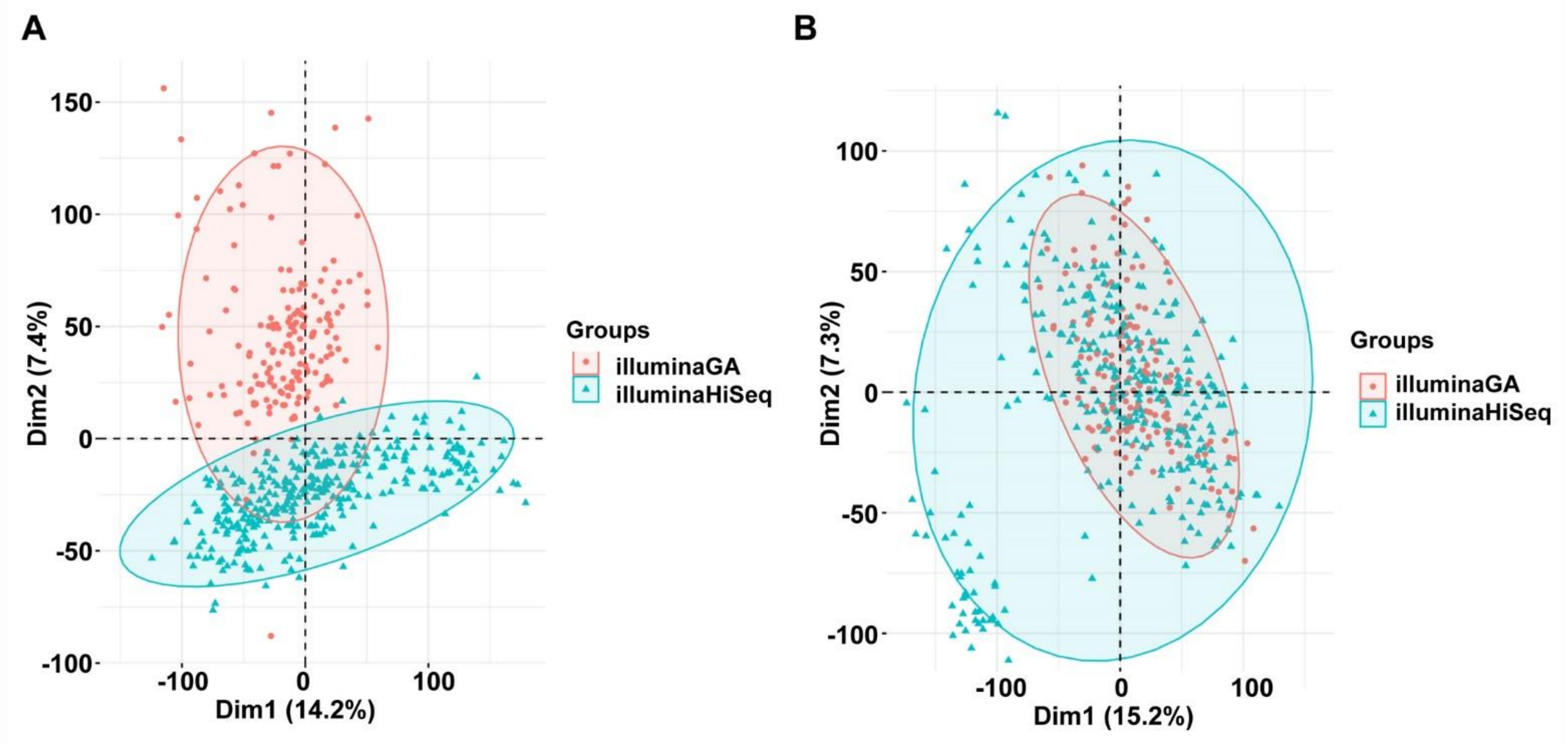

Supplementary Figure S1 Principal component analysis (PCA) of the two colon cancer data sets from TCGA. The points of the scatter plots visualize the samples based on two platform illuminiaGA and illuminiaHiSeq without **(A)** and with **(B)** the removal of the batch effect.

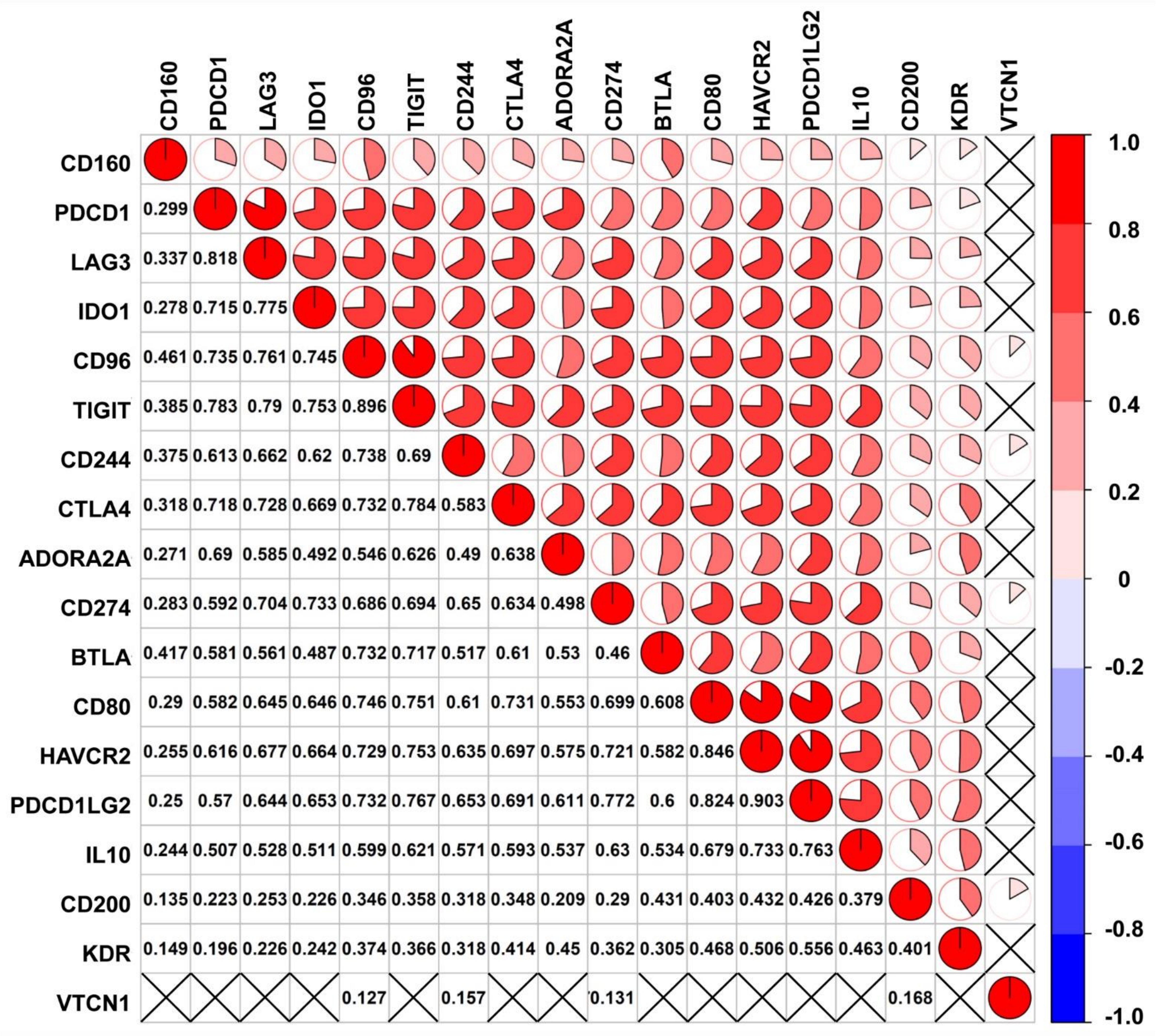

Supplementary Figure S2 The correlation plot for immune-resistance markers using the TCGA COAD cohort. The upper right triangle represents the degree of correlation degree by combining the size and color of the pie. The lower left triangle shows specific Spearman correlation coefficients for every pair of genes. The corresponding *P* value, which was not considered significant, is here marked with a cross (*P* > 0.01).

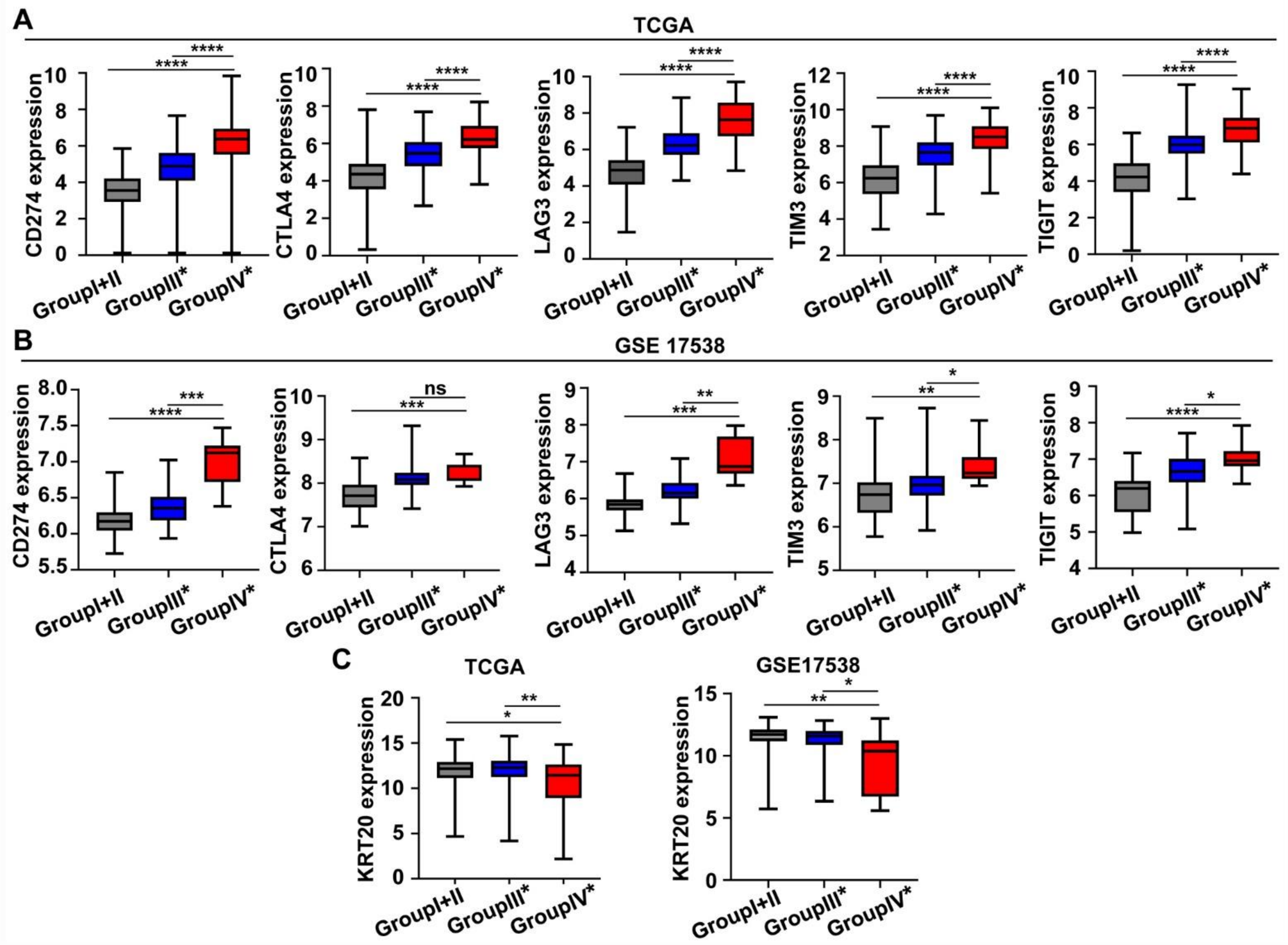

Supplementary Figure S3 Comparison of the levels of expression of representative immune checkpoint genes across the colon cancer risk groups from the **(A)** TCGA and **(B)** GEO GSE17538 data sets. **(C)** KRT20 (the cancer cell marker) mRNA expression levels were compared across the colon cancer risk groups from the TCGA and GEO GSE17538 data sets. ****, $P < 0.0001$; ***, $P < 0.001$; **, $P < 0.01$; *, $P < 0.05$; ns, not significant.

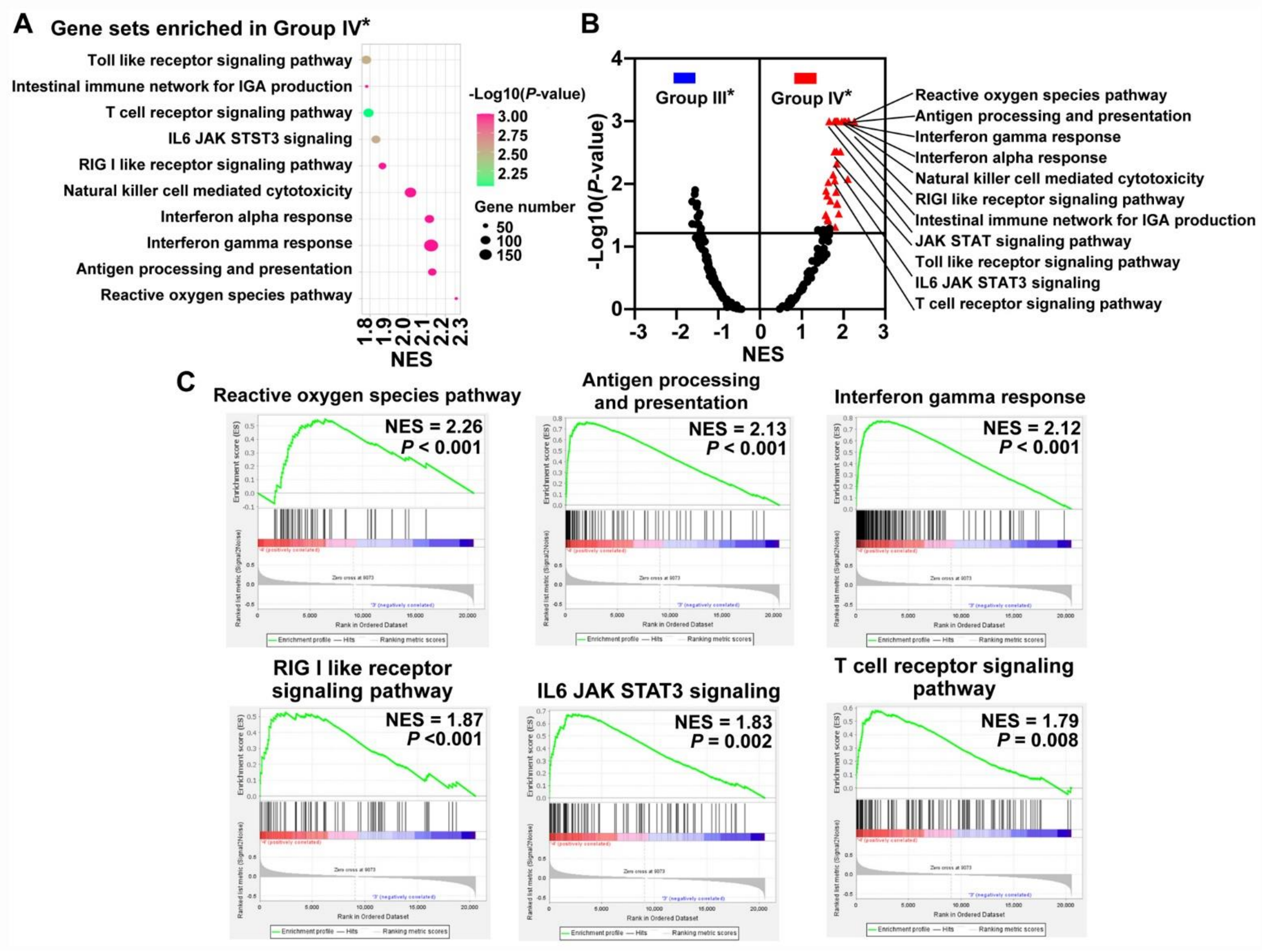

Supplementary Figure S4 Different immune statuses between Group IV* and Group III* by functional and pathway enrichment analysis. The pathways enriched in Group IV* predicted by GSEA were visualized by a **(A)** bubble plot and **(B)** volcano plot. **(C)** GSEA results showing significant enrichment of immune-related phenotypes, including the reactive oxygen species pathway, interferon gamma response, interferon alpha response, antigen processing and presentation, toll-like receptor signaling pathway, natural-killer-cell-mediated cytotoxicity, RIGI-like receptor signaling pathway, IL6 JAK STST3 signaling pathway, and T cell receptor signaling pathway in colon cancer patients in Group IV* relative to Group III*. NES, normalized enrichment score.

Supplementary Table S1 The correlation coefficient (r) between IDO1 and TILs, and the corresponding *P* value across human heterogeneous cancers from the TISIDB database.*

Cancer type	r/*P*	Act CD8	Tcm CD8	Tem CD8	Act CD4	Tcm CD4	Tem CD4	Tfh	Tgd	Th1	Th17	Th2	Treg	Act B	Imm B
ACC (n=79)	r	0.188	0.205	0.127	0.133	0.066	0.136	0.289	0.255	0.089	0.011	0.019	0.271	0.255	0.222
	P	0.097	0.070	0.266	0.241	0.563	0.231	0.010	0.024	0.435	0.921	0.865	0.016	0.046	0.049
BLCA (n=408)	r	0.699	0.444	0.725	0.627	0.503	0.220	0.583	0.451	0.648	0.300	0.528	0.622	0.513	0.607
	P	0.000	0.000	0.000	0.000	0.000	0.018	0.000	0.000	0.000	0.000	0.000	0.000	0.000	0.000
BRCA (n=1100)	r	0.754	0.200	0.706	0.738	0.382	0.357	0.677	0.533	0.648	0.413	0.439	0.570	0.630	0.681
	P	0.000	0.000	0.000	0.000	0.000	0.000	0.000	0.000	0.000	0.000	0.000	0.000	0.000	0.000
CESC (n=306)	r	0.606	0.156	0.693	0.549	0.409	0.261	0.457	0.396	0.522	0.210	0.377	0.466	0.456	0.611
	P	0.000	0.006	0.000	0.000	0.000	0.010	0.000	0.000	0.000	0.000	0.000	0.000	0.000	0.000
CHOL (n=36)	r	0.683	0.289	0.814	0.699	0.366	0.595	0.743	0.395	0.821	0.376	0.575	0.720	0.783	0.765
	P	0.020	0.088	0.001	0.009	0.029	0.000	0.008	0.018	0.001	0.024	0.000	0.005	0.003	0.005
COAD (n=459)	r	0.710	0.389	0.730	0.539	0.461	0.358	0.664	0.464	0.666	0.294	0.479	0.586	0.509	0.615
	P	0.000	0.000	0.000	0.000	0.000	0.000	0.000	0.000	0.000	0.000	0.000	0.000	0.000	0.000
ESCA (n=185)	r	0.736	0.211	0.680	0.488	0.213	0.271	0.551	0.249	0.585	0.343	0.311	0.413	0.546	0.678
	P	0.000	0.004	0.000	0.000	0.004	0.000	0.000	0.001	0.000	0.005	0.012	0.001	0.000	0.000
GBM (n=166)	r	0.514	0.290	0.582	0.179	0.392	0.351	0.459	0.422	0.535	0.124	0.005	0.347	0.419	0.326
	P	0.000	0.000	0.000	0.021	0.002	0.010	0.000	0.001	0.000	0.111	0.952	0.013	0.001	0.014
HNSC (n=522)	r	0.725	0.227	0.757	0.587	0.252	0.288	0.512	0.304	0.569	0.407	0.411	0.471	0.528	0.639
	P	0.000	0.002	0.000	0.000	0.001	0.000	0.000	0.000	0.000	0.000	0.000	0.000	0.000	0.000
KICH (n=66)	r	0.099	-0.038	0.001	0.074	-0.050	0.065	-0.039	-0.036	-0.130	0.047	-0.112	-0.133	0.023	-0.083
	P	0.428	0.763	0.993	0.555	0.691	0.601	0.757	0.775	0.298	0.709	0.369	0.286	0.857	0.505
KIRC (n=534)	r	0.275	0.034	0.369	0.196	0.176	0.249	0.257	0.167	0.240	0.061	0.140	0.176	0.203	0.218
	P	0.000	0.430	0.000	0.013	0.030	0.001	0.000	0.000	0.001	0.156	0.001	0.030	0.006	0.003
KIRP (n=291)	r	0.229	0.157	0.118	0.428	0.104	0.253	0.137	0.227	0.130	-0.133	0.218	0.259	0.150	0.127
	P	0.055	0.007	0.044	0.000	0.076	0.009	0.020	0.067	0.026	0.024	0.000	0.021	0.010	0.030
LGG (n=530)	r	0.576	0.450	0.590	0.398	0.500	0.111	0.463	0.541	0.477	0.203	0.282	0.520	0.374	0.370
	P	0.000	0.000	0.000	0.000	0.000	0.010	0.000	0.000	0.000	0.006	0.000	0.000	0.000	0.000
LIHC (n=373)	r	0.496	0.217	0.504	0.387	0.258	0.310	0.467	0.372	0.360	0.149	0.286	0.389	0.501	0.501
	P	0.000	0.017	0.000	0.000	0.005	0.000	0.000	0.000	0.000	0.004	0.001	0.000	0.000	0.000
LUAD (n=517)	r	0.583	0.227	0.527	0.492	0.232	0.243	0.408	0.260	0.462	0.199	0.342	0.407	0.405	0.431
	P	0.000	0.002	0.000	0.000	0.001	0.001	0.000	0.000	0.000	0.013	0.000	0.000	0.000	0.000
LUSC (n=501)	r	0.618	0.177	0.550	0.494	0.325	0.287	0.395	0.338	0.479	0.354	0.323	0.399	0.460	0.507
	P	0.000	0.046	0.000	0.000	0.000	0.000	0.000	0.000	0.000	0.000	0.000	0.000	0.000	0.000
MESO (n=87)	r	0.667	0.262	0.550	0.372	0.430	0.182	0.553	0.419	0.500	0.308	0.258	0.528	0.407	0.448
	P	0.000	0.015	0.002	0.000	0.026	0.091	0.002	0.042	0.003	0.004	0.016	0.002	0.000	0.012
OV (n=307)	r	0.585	0.335	0.550	0.397	0.338	0.296	0.351	0.203	0.432	0.426	0.148	0.390	0.350	0.477
	P	0.000	0.000	0.000	0.000	0.000	0.001	0.000	0.000	0.000	0.000	0.009	0.000	0.000	0.000
PAAD (n=179)	r	0.554	0.227	0.650	0.519	0.171	0.445	0.524	0.329	0.532	0.050	0.379	0.468	0.485	0.580
	P	0.000	0.002	0.000	0.000	0.022	0.000	0.000	0.019	0.000	0.506	0.002	0.000	0.000	0.000
PCPG (n=184)	r	0.313	0.226	0.170	0.195	0.304	0.310	0.301	0.264	0.224	0.134	0.138	0.219	0.191	0.177
	P	0.012	0.002	0.021	0.008	0.020	0.014	0.025	0.000	0.002	0.070	0.061	0.003	0.009	0.016
PRAD (n=498)	r	0.580	0.264	0.480	0.531	0.362	0.267	0.410	0.242	0.461	0.223	0.340	0.440	0.479	0.521
	P	0.000	0.000	0.000	0.000	0.000	0.000	0.000	0.002	0.000	0.005	0.000	0.000	0.000	0.000
READ (n=167)	r	0.635	0.281	0.649	0.561	0.297	0.400	0.658	0.475	0.635	0.193	0.384	0.574	0.532	0.618
	P	0.000	0.000	0.000	0.000	0.000	0.001	0.000	0.000	0.000	0.013	0.003	0.000	0.000	0.000
SARC (n=263)	r	0.577	0.289	0.586	0.385	0.281	0.352	0.581	0.326	0.517	0.345	0.226	0.471	0.606	0.632
	P	0.000	0.005	0.000	0.000	0.010	0.001	0.000	0.003	0.000	0.000	0.000	0.000	0.000	0.000
SKCM (n=472)	r	0.806	0.217	0.794	0.621	0.414	0.400	0.698	0.360	0.715	0.561	0.340	0.673	0.730	0.758
	P	0.000	0.005	0.000	0.000	0.000	0.000	0.000	0.000	0.000	0.000	0.000	0.000	0.000	0.000
STAD (n=415)	r	0.737	0.353	0.635	0.696	0.434	0.245	0.530	0.367	0.532	0.206	0.378	0.477	0.423	0.530
	P	0.000	0.000	0.000	0.000	0.000	0.005	0.000	0.000	0.000	0.017	0.000	0.000	0.000	0.000
TGCT (n=156)	r	0.757	0.226	0.717	0.732	0.507	0.465	0.738	0.182	0.608	0.384	0.075	0.532	0.634	0.675
	P	0.000	0.005	0.000	0.000	0.000	0.000	0.000	0.023	0.000	0.008	0.350	0.000	0.000	0.000
THCA (n=509)	r	0.641	0.332	0.630	0.571	0.406	0.378	0.568	0.473	0.627	0.282	0.402	0.555	0.636	0.605
	P	0.000	0.000	0.000	0.000	0.000	0.000	0.000	0.000	0.000	0.000	0.000	0.000	0.000	0.000
UCEC (n=546)	r	0.298	0.203	0.503	0.266	0.307	0.140	0.362	0.213	0.451	0.335	0.165	0.344	0.391	0.422
	P	0.000	0.005	0.000	0.000	0.000	0.001	0.000	0.005	0.000	0.000	0.000	0.000	0.000	0.000
UCS (n=57)	r	0.577	0.227	0.664	0.396	0.511	0.202	0.604	0.290	0.526	0.544	-0.061	0.595	0.504	0.623
	P	0.010	0.089	0.002	0.002	0.042	0.131	0.003	0.029	0.023	0.012	0.651	0.004	0.055	0.004
UVM (n=80)	r	0.741	0.348	0.722	0.598	0.467	0.406	0.624	0.397	0.740	0.428	0.260	0.645	0.744	0.678
	P	0.000	0.002	0.000	0.001	0.011	0.000	0.000	0.000	0.000	0.058	0.020	0.000	0.000	0.000

Cancer type	r/*P*	Mem B	NK	CD56bright	CD56dim	MDSC	NKT	Act DC	pDC	iDC	Macrophage	Eosinophil	Mast	Monocyte	Neutrophil
ACC (n=79)	r	-0.038	0.189	0.258	0.113	0.281	0.346	0.205	-0.045	0.165	0.286	0.195	0.187	0.319	0.267
	P	0.737	0.095	0.022	0.321	0.012	0.002	0.070	0.691	0.145	0.011	0.085	0.099	0.004	0.018
BLCA (n=408)	r	0.243	0.540	0.311	0.263	0.633	0.623	0.636	0.325	0.249	0.521	0.385	0.346	0.298	0.501
	P	0.007	0.000	0.000	0.003	0.000	0.000	0.000	0.000	0.004	0.000	0.000	0.000	0.000	0.000
BRCA (n=1100)	r	0.269	0.392	0.503	0.344	0.672	0.608	0.618	0.182	0.110	0.484	0.362	0.418	0.418	0.151
	P	0.000	0.000	0.000	0.000	0.000	0.000	0.000	0.000	0.000	0.000	0.000	0.000	0.000	0.005
CESC (n=306)	r	0.051	0.485	0.381	0.209	0.567	0.561	0.534	0.153	0.342	0.419	0.395	0.335	0.386	0.256
	P	0.378	0.000	0.000	0.000	0.000	0.000	0.000	0.007	0.000	0.000	0.000	0.000	0.000	0.015
CHOL (n=36)	r	0.537	0.529	0.314	0.072	0.764	0.816	0.498	0.245	0.186	0.766	0.642	0.684	-0.227	0.275
	P	0.001	0.001	0.062	0.677	0.005	0.001	0.002	0.149	0.275	0.005	0.025	0.019	0.183	0.105
COAD (n=459)	r	0.051	0.517	0.268	0.203	0.649	0.590	0.622	0.377	0.345	0.521	0.360	0.449	0.223	0.430
	P	0.274	0.000	0.001	0.009	0.000	0.000	0.000	0.000	0.000	0.000	0.000	0.000	0.004	0.000
ESCA (n=185)	r	0.274	0.528	0.174	0.224	0.601	0.466	0.512	0.210	0.059	0.452	0.408	0.395	0.329	0.330
	P	0.000	0.000	0.018	0.002	0.000	0.000	0.000	0.004	0.421	0.000	0.000	0.001	0.013	0.012
GBM (n=166)	r	-0.027	0.374	0.194	0.284	0.522	0.384	0.501	0.429	0.322	0.420	0.222	0.392	0.371	0.217
	P	0.732	0.008	0.012	0.000	0.000	0.004	0.000	0.000	0.017	0.001	0.004	0.002	0.003	0.005
HNSC (n=522)	r	0.126	0.472	0.325	0.097	0.641	0.493	0.550	0.064	0.239	0.419	0.417	0.290	0.370	0.208
	P	0.004	0.000	0.000	0.026	0.000	0.000	0.000	0.143	0.001	0.000	0.000	0.000	0.000	0.004
KICH (n=66)	r	-0.123	-0.074	-0.014	0.083	0.060	-0.048	-0.023	0.209	-0.226	-0.082	0.155	-0.039	0.088	0.051
	P	0.325	0.553	0.909	0.504	0.631	0.701	0.852	0.092	0.068	0.513	0.213	0.755	0.483	0.683
KIRC (n=534)	r	0.036	0.249	0.229	0.122	0.225	0.269	0.045	0.185	0.103	0.154	0.169	0.134	0.122	0.154
	P	0.412	0.001	0.003	0.005	0.002	0.000	0.299	0.012	0.017	0.000	0.062	0.002	0.005	0.000
KIRP (n=291)	r	0.142	0.087	0.138	0.143	0.122	0.190	0.148	0.074	0.106	0.104	0.147	0.019	0.035	0.093
	P	0.016	0.140	0.019	0.015	0.037	0.001	0.012	0.211	0.072	0.076	0.012	0.752	0.555	0.114
LGG (n=530)	r	0.344	0.380	0.314	0.299	0.558	0.492	0.527	0.275	0.308	0.415	0.081	0.488	0.294	0.411
	P	0.000	0.000	0.000	0.000	0.000	0.000	0.000	0.000	0.000	0.000	0.061	0.000	0.000	0.000
LIHC (n=373)	r	0.164	0.299	0.269	0.117	0.443	0.339	0.299	0.232	0.191	0.358	0.325	0.373	0.165	0.204
	P	0.002	0.001	0.001	0.024	0.000	0.000	0.001	0.016	0.000	0.000	0.000	0.000	0.001	0.052
LUAD (n=517)	r	0.276	0.347	0.422	0.199	0.461	0.467	0.398	0.179	0.022	0.299	0.148	0.193	0.241	0.199
	P	0.000	0.000	0.000	0.014	0.000	0.000	0.000	0.030	0.625	0.000	0.001	0.007	0.001	0.013
LUSC (n=501)	r	0.141	0.404	0.275	0.167	0.524	0.364	0.466	0.222	0.197	0.428	0.337	0.364	0.344	0.334
	P	0.002	0.000	0.000	0.000	0.000	0.000	0.000	0.005	0.023	0.000	0.000	0.000	0.000	0.000
MESO (n=87)	r	0.026	0.457	0.327	0.266	0.604	0.516	0.545	0.208	0.188	0.407	0.247	0.251	0.416	0.281
	P	0.812	0.007	0.002	0.013	0.000	0.004	0.003	0.053	0.081	0.000	0.021	0.019	0.048	0.009
OV (n=307)	r	0.058	0.375	0.167	0.314	0.473	0.343	0.490	0.261	0.200	0.305	0.201	0.351	0.342	0.286
	P	0.314	0.000	0.003	0.001	0.000	0.000	0.000	0.009	0.000	0.002	0.000	0.000	0.000	0.004
PAAD (n=179)	r	0.419	0.478	0.085	0.066	0.539	0.517	0.338	0.229	0.161	0.467	0.350	0.467	0.204	0.328
	P	0.001	0.000	0.255	0.382	0.000	0.000	0.011	0.002	0.032	0.000	0.005	0.000	0.006	0.020
PCPG (n=184)	r	-0.083	0.176	0.164	0.104	0.301	0.222	0.259	0.094	0.136	0.233	0.154	0.197	0.285	0.101
	P	0.261	0.017	0.026	0.159	0.024	0.003	0.000	0.204	0.065	0.001	0.037	0.008	0.065	0.174
PRAD (n=498)	r	0.260	0.340	0.238	0.210	0.513	0.364	0.486	0.111	0.141	0.323	0.338	0.282	0.093	0.236
	P	0.001	0.000	0.003	0.006	0.000	0.000	0.000	0.013	0.002	0.000	0.000	0.000	0.037	0.001
READ (n=167)	r	0.247	0.411	0.211	0.025	0.607	0.549	0.525	0.276	0.298	0.515	0.471	0.492	0.084	0.378
	P	0.001	0.001	0.006	0.751	0.000	0.000	0.000	0.000	0.065	0.000	0.000	0.000	0.280	0.005
SARC (n=263)	r	0.230	0.600	0.316	0.098	0.525	0.469	0.413	0.258	0.325	0.460	0.378	0.412	0.417	0.510
	P	0.000	0.000	0.002	0.113	0.000	0.000	0.000	0.016	0.003	0.000	0.000	0.000	0.000	0.000
SKCM (n=472)	r	0.384	0.666	0.307	0.148	0.730	0.702	0.653	0.180	0.339	0.648	0.584	0.523	0.338	0.406
	P	0.000	0.000	0.000	0.001	0.000	0.000	0.000	0.059	0.000	0.000	0.000	0.000	0.000	0.000
STAD (n=415)	r	0.342	0.478	0.338	0.248	0.587	0.467	0.549	0.249	0.065	0.392	0.218	0.253	0.333	0.165
	P	0.000	0.000	0.000	0.003	0.000	0.000	0.000	0.003	0.183	0.000	0.020	0.002	0.000	0.001
TGCT (n=156)	r	0.351	0.476	0.476	0.550	0.730	0.674	0.623	0.407	0.122	0.548	0.510	0.422	0.509	0.450
	P	0.020	0.000	0.000	0.000	0.000	0.000	0.000	0.002	0.130	0.000	0.000	0.002	0.000	0.001
THCA (n=509)	r	0.453	0.447	0.414	0.307	0.616	0.579	0.558	0.184	0.099	0.514	0.297	0.404	0.190	0.319
	P	0.000	0.000	0.000	0.000	0.000	0.000	0.000	0.021	0.026	0.000	0.000	0.000	0.011	0.000
UCEC (n=546)	r	0.041	0.365	0.212	0.205	0.373	0.266	0.421	0.181	0.186	0.350	0.218	0.218	0.183	0.314
	P	0.338	0.000	0.006	0.004	0.000	0.000	0.000	0.014	0.008	0.000	0.002	0.002	0.012	0.000
UCS (n=57)	r	0.306	0.579	0.354	0.231	0.683	0.227	0.627	0.506	0.506	0.643	0.275	0.520	0.507	0.292
	P	0.021	0.009	0.007	0.083	0.000	0.090	0.003	0.049	0.050	0.002	0.039	0.030	0.049	0.028
UVM (n=80)	r	0.223	0.590	0.440	0.362	0.648	0.518	0.671	0.166	0.414	0.518	0.275	0.612	0.126	0.608
	P	0.047	0.001	0.035	0.001	0.000	0.003	0.000	0.142	0.000	0.003	0.014	0.000	0.265	0.000

*The cells with gray background represent the *P* value is less than 0.05, which is considered insignificant.

Supplementary Table S2 The correlation coefficient (r) between IDO1 and CD8 T cells in TILs, and the corresponding *P* value in the pan-cancer profiling from the TIMER2.0 database.*

Cancer type	r/*P*	T cell CD8+ central memory XCELL	T cell CD8+ effector memory XCELL	T cell CD8+ naive XCELL	T cell CD8+ CIBERSORT	T cell CD8+ CIBERSORT-ABS	T cell CD8+ EPIC	T cell CD8+ MCPCOUNTER	T cell CD8+ QUANTISEQ	T cell CD8+ TIMER	T cell CD8+ XCELL
ACC (n=79)	r	-0.007	0.278	0.015	-0.098	-0.031	-0.083	-0.085	-0.121	-0.168	0.012
	P	0.951	0.017	0.901	0.411	0.794	0.487	0.476	0.310	0.156	0.918
BLCA (n=408)	r	0.592	0.242	0.060	0.434	0.593	-0.066	0.483	0.503	0.330	0.332
	P	0.000	0.000	0.248	0.000	0.000	0.208	0.000	0.000	0.000	0.000
BRCA (n=1100)	r	0.676	0.132	0.254	0.346	0.567	0.264	0.633	0.663	0.058	0.543
	P	0.000	0.000	0.000	0.000	0.000	0.000	0.000	0.000	0.066	0.000
BRCA-Basal (n=191)	r	0.689	0.337	0.262	0.323	0.515	0.329	0.500	0.564	0.076	0.526
	P	0.000	0.000	0.000	0.000	0.000	0.000	0.000	0.000	0.321	0.000
BRCA-Her2 (n=82)	r	0.714	0.153	0.171	0.526	0.693	0.316	0.708	0.713	0.149	0.564
	P	0.000	0.198	0.150	0.000	0.000	0.007	0.000	0.000	0.211	0.000
BRCA-LumA (n=568)	r	0.704	0.012	0.309	0.368	0.583	0.228	0.652	0.692	0.209	0.500
	P	0.000	0.782	0.000	0.000	0.000	0.000	0.000	0.000	0.000	0.000
BRCA-LumB (n=219)	r	0.495	0.112	0.228	0.367	0.562	0.199	0.621	0.673	0.116	0.470
	P	0.000	0.123	0.001	0.000	0.000	0.006	0.000	0.000	0.109	0.000
CESC (n=306)	r	0.614	0.369	0.118	0.460	0.596	0.364	0.466	0.551	0.581	0.516
	P	0.000	0.000	0.049	0.000	0.000	0.000	0.000	0.000	0.000	0.000
CHOL (n=36)	r	0.726	0.100	0.293	0.470	0.648	0.349	0.500	0.505	0.483	0.567
	P	0.000	0.567	0.088	0.004	0.000	0.040	0.002	0.002	0.003	0.000
COAD (n=458)	r	0.668	0.211	0.021	0.420	0.704	0.016	0.373	0.533	0.494	0.445
	P	0.000	0.000	0.734	0.000	0.000	0.786	0.000	0.000	0.000	0.000
DLBC (n=48)	r	0.611	0.557	-0.066	0.396	0.702	0.270	0.458	0.562	0.569	0.450
	P	0.000	0.000	0.681	0.010	0.000	0.088	0.003	0.000	0.000	0.003
ESCA (n=185)	r	0.634	0.234	0.133	0.490	0.652	0.062	0.680	0.708	0.473	0.384
	P	0.000	0.002	0.074	0.000	0.000	0.405	0.000	0.000	0.000	0.000
GBM (n=153)	r	0.259	-0.060	-0.019	0.057	0.128	-0.120	0.360	0.071	0.274	-0.065
	P	0.002	0.484	0.825	0.505	0.136	0.164	0.000	0.409	0.001	0.448
HNSC (n=522)	r	0.753	0.503	0.163	0.573	0.697	0.262	0.726	0.736	0.116	0.613
	P	0.000	0.000	0.000	0.000	0.000	0.000	0.000	0.000	0.010	0.000
HNSC-HPV- (n=422)	r	0.724	0.502	0.175	0.528	0.666	0.163	0.706	0.723	0.204	0.612
	P	0.000	0.000	0.000	0.000	0.000	0.001	0.000	0.000	0.000	0.000
HNSC-HPV+ (n=98)	r	0.715	0.445	0.087	0.583	0.678	0.526	0.641	0.650	-0.018	0.533
	P	0.000	0.000	0.419	0.000	0.000	0.000	0.000	0.000	0.866	0.000
KICH (n=66)	r	0.122	0.046	0.056	0.054	0.104	0.032	0.160	-0.026	0.098	-0.027
	P	0.334	0.718	0.659	0.670	0.408	0.799	0.203	0.840	0.436	0.830
KIRC (n=533)	r	0.304	0.299	0.297	0.211	0.328	0.293	0.331	0.287	0.414	0.288
	P	0.000	0.000	0.000	0.000	0.000	0.000	0.000	0.000	0.000	0.000
KIRP (n=290)	r	0.163	0.126	-0.091	0.054	0.149	0.058	0.165	0.259	0.223	0.104
	P	0.009	0.044	0.146	0.391	0.017	0.355	0.008	0.000	0.000	0.096
LGG (n=516)	r	-0.110	-0.038	-0.032	0.226	0.324	-0.170	0.217	0.167	-0.265	0.037
	P	0.016	0.407	0.479	0.000	0.000	0.000	0.000	0.000	0.000	0.416
LIHC (n=371)	r	0.437	0.184	0.180	0.258	0.416	0.068	0.452	0.411	0.463	0.413
	P	0.000	0.001	0.001	0.000	0.000	0.205	0.000	0.000	0.000	0.000
LUAD (n=515)	r	0.475	0.218	0.081	0.250	0.429	0.015	0.492	0.493	0.395	0.373
	P	0.000	0.000	0.073	0.000	0.000	0.747	0.000	0.000	0.000	0.000
LUSC (n=501)	r	0.608	0.381	0.116	0.422	0.541	0.257	0.514	0.555	0.309	0.533
	P	0.000	0.000	0.011	0.000	0.000	0.000	0.000	0.000	0.000	0.000
MESO (n=87)	r	0.665	0.405	0.163	0.487	0.585	0.300	0.583	0.625	0.393	0.513
	P	0.000	0.000	0.136	0.000	0.000	0.005	0.000	0.000	0.000	0.000
OV (n=303)	r	0.430	0.157	-0.164	0.253	0.346	-0.108	0.246	0.315	0.335	0.173
	P	0.000	0.013	0.009	0.000	0.000	0.088	0.000	0.000	0.000	0.006
PAAD (n=179)	r	0.607	0.164	0.221	0.324	0.623	0.102	0.589	0.632	0.587	0.543
	P	0.000	0.032	0.004	0.000	0.000	0.185	0.000	0.000	0.000	0.000

Cancer type	r/*P*	T cell CD8+ central memory XCELL	T cell CD8+ effector memory XCELL	T cell CD8+ naive XCELL	T cell CD8+ CIBERSORT	T cell CD8+ CIBERSORT-ABS	T cell CD8+ EPIC	T cell CD8+ MCPCOUNTER	T cell CD8+ QUANTISEQ	T cell CD8+ TIMER	T cell CD8+ XCELL
PCPG (n=181)	r	0.047	0.096	-0.079	-0.133	0.050	-0.120	0.070	0.067	0.053	-0.005
	P	0.545	0.216	0.307	0.087	0.524	0.122	0.368	0.391	0.497	0.945
PRAD (n=498)	r	0.628	0.093	-0.036	0.135	0.524	0.186	0.443	0.467	0.083	0.345
	P	0.000	0.057	0.460	0.006	0.000	0.000	0.000	0.000	0.092	0.000
READ (n=166)	r	0.374	0.326	-0.009	0.186	0.540	-0.080	0.268	0.414	0.335	0.315
	P	0.000	0.002	0.930	0.080	0.000	0.454	0.011	0.000	0.001	0.002
SARC (n=260)	r	0.456	0.307	0.118	0.376	0.521	0.262	0.564	0.449	0.173	0.357
	P	0.000	0.000	0.067	0.000	0.000	0.000	0.000	0.000	0.007	0.000
SKCM (n=471)	r	0.704	0.271	0.318	0.502	0.740	0.452	0.759	0.744	0.341	0.628
	P	0.000	0.000	0.000	0.000	0.000	0.000	0.000	0.000	0.000	0.000
SKCM-Metastasis (n=368)	r	0.696	0.284	0.358	0.540	0.740	0.476	0.745	0.741	0.297	0.633
	P	0.000	0.000	0.000	0.000	0.000	0.000	0.000	0.000	0.000	0.000
SKCM-Primary (n=103)	r	0.620	0.145	0.316	0.458	0.633	0.331	0.692	0.637	0.416	0.508
	P	0.000	0.147	0.001	0.000	0.000	0.001	0.000	0.000	0.000	0.000
STAD (n=415)	r	0.642	0.461	0.243	0.416	0.612	0.176	0.559	0.622	0.663	0.508
	P	0.000	0.000	0.000	0.000	0.000	0.001	0.000	0.000	0.000	0.000
TGCT (n=150)	r	0.545	0.479	0.008	-0.004	0.316	-0.060	0.348	0.279	0.477	0.385
	P	0.000	0.000	0.925	0.959	0.000	0.471	0.000	0.001	0.000	0.000
THCA (n=509)	r	0.544	0.249	-0.106	0.216	0.594	-0.081	0.460	0.526	-0.454	0.431
	P	0.000	0.000	0.019	0.000	0.000	0.076	0.000	0.000	0.000	0.000
THYM (n=120)	r	0.514	0.235	-0.127	0.086	0.545	-0.052	-0.070	-0.032	-0.181	0.204
	P	0.000	0.012	0.177	0.363	0.000	0.580	0.458	0.731	0.053	0.029
UCEC (n=545)	r	0.315	0.100	-0.023	0.135	0.311	0.053	0.257	0.290	0.068	0.138
	P	0.003	0.352	0.834	0.210	0.003	0.623	0.016	0.006	0.531	0.200
UCS (n=57)	r	0.261	-0.006	-0.245	0.029	0.272	0.048	0.322	0.293	0.041	0.229
	P	0.059	0.964	0.077	0.837	0.049	0.731	0.019	0.033	0.771	0.099
UVM (n=80)	r	0.623	0.189	0.304	0.584	0.706	0.319	0.791	0.821	0.294	0.500
	P	0.000	0.100	0.007	0.000	0.000	0.005	0.000	0.000	0.009	0.000

*The cells with gray background represent the *P* value is less than 0.05, which is considered insignificant.
